# Sugarcane (*Saccharum officinarum* L.) Top Extract Ameliorates Cognitive Decline in Senescence Model SAMP8 Mice: Modulation of Neural Development and Energy Metabolism

**DOI:** 10.3389/fcell.2020.573487

**Published:** 2020-10-06

**Authors:** Kengo Iwata, Qingqing Wu, Farhana Ferdousi, Kazunori Sasaki, Kenichi Tominaga, Haruhisa Uchida, Yoshinobu Arai, Francis G. Szele, Hiroko Isoda

**Affiliations:** ^1^School of Integrative and Global Majors, University of Tsukuba, Tsukuba, Japan; ^2^Nippo Co., Ltd., Daito, Japan; ^3^Alliance for Research on the Mediterranean and North Africa, University of Tsukuba, Tsukuba, Japan; ^4^Department of Physiology, Anatomy and Genetics, University of Oxford, Oxford, United Kingdom; ^5^AIST-University of Tsukuba Open Innovation Laboratory for Food and Medicinal Resource Engineering (FoodMed-OIL), AIST, University of Tsukuba, Tsukuba, Japan; ^6^Faculty of Life and Environmental Sciences, University of Tsukuba, Tsukuba, Japan

**Keywords:** cognitive function, sugarcane top, polyphenol, energy metabolism, neural development, SAMP8

## Abstract

Age-related biological alterations in brain function increase the risk of mild cognitive impairment and dementia, a global problem exacerbated by aging populations in developed nations. Limited pharmacological therapies have resulted in attention turning to the promising role of medicinal plants and dietary supplements in the treatment and prevention of dementia. Sugarcane (*Saccharum officinarum* L.) top, largely considered as a by-product because of its low sugar content, in fact contains the most abundant amounts of antioxidant polyphenols relative to the rest of the plant. Given the numerous epidemiological studies on the effects of polyphenols on cognitive function, in this study, we analyzed polyphenolic constituents of sugarcane top and examined the effect of sugarcane top ethanolic extract (STEE) on a range of central nervous system functions *in vitro* and *in vivo*. Orally administrated STEE rescued spatial learning and memory deficit in the senescence-accelerated mouse prone 8 (SAMP8) mice, a non-transgenic strain that spontaneously develops a multisystemic aging phenotype including pathological features of Alzheimer’s disease. This could be correlated with an increased number of hippocampal newborn neurons and restoration of cortical monoamine levels in STEE-fed SAMP8 mice. Global genomic analysis by microarray in cerebral cortices showed multiple potential mechanisms for the cognitive improvement. Gene set enrichment analysis (GSEA) revealed biological processes such as neurogenesis, neuron differentiation, and neuron development were significantly enriched in STEE-fed mice brain compared to non-treated SAMP8 mice. Furthermore, STEE treatment significantly regulated genes involved in neurotrophin signaling, glucose metabolism, and neural development in mice brain. Our *in vitro* results suggest that STEE treatment enhances the metabolic activity of neuronal cells promoting glucose metabolism with significant upregulation of genes, namely *PGK1*, *PGAM1*, *PKM*, and *PC*. STEE also stimulated proliferation of human neural stem cells (hNSCs), regulated bHLH factor expression and induced neuronal differentiation and astrocytic process lengthening. Altogether, our findings suggest the potential of STEE as a dietary intervention, with promising implications as a novel nutraceutical for cognitive health.

## Introduction

Cognitive decline, such as memory loss and learning deficit, is highly associated with aging. This has been implicated to be mediated by oxidative stress, mitochondrial dysfunction, and defective apoptotic processes in the process of normal brain aging (Ginneken, 2017). In addition, other protein abnormalities such as increased amyloid-β (Aβ) plaques, phosphorylated tau and neurofibrillary tangles, and Lewy bodies are the pathological hallmarks of severe brain disorders, including Alzheimer’s disease (AD). Age-related brain disorders are generally accompanied by other pathologies, including cortical (particularly hippocampal) shrinkage, abnormal immune response and neurogenesis, and decreased neurotransmitter concentration, which could theoretically be pharmacological targets (Nelson and Tabet, 2015; Poulose et al., 2017). With the rapid growth of the aging population and increasing awareness of the risk of dementia, prevention and treatment of age-related cognitive decline have become research priorities. Pharmacological therapies are currently limited, and recently attention has turned to the role of dietary interventions or nutraceuticals in this respect (Williams et al., 2011; Howes et al., 2020).

Sugarcane (*Saccharum officinarum* L.) is one of the most widely distributed plants in subtropical and tropical regions and is cultivated as an important source of sugar. From the industrial point of view, the most profitable part of the plant is the stem, which contains relatively high concentrations of sugar. In contrast, the top part of sugarcane including leaves (sugarcane top) are often treated as waste, although some of them are used in fermented silage. However, it has been reported that sugarcane top in fact contains a larger amount of polyphenol with antioxidant properties compared to its stem (Colombo et al., 2005, 2006; Sun et al., 2014).

Caffeoylquinic acid (CQA), also known as chlorogenic acid, is a naturally occurring antioxidant polyphenol found in various plants. CQA derivatives have been shown to have neuroprotective effects and to improve learning and memory via inhibition of Aβ aggregation and enhancement of ATP production both *in vitro* and *in vivo* (Han et al., 2010; Miyamae et al., 2011b, 2012). Furthermore, past studies have shown that CQA-rich plant extracts can ameliorate aging-related cognitive deficit, depressive behavior, and amyloid pathologies *in vivo* (Sasaki et al., 2013; Wu et al., 2016; Lim et al., 2018; Ishida et al., 2020). Moreover, our recent study suggested that intracorporeal mono-CQAs, degraded from 3,4,5-tricaffeoylquinic acid, stimulate hippocampal neurogenesis (Sasaki et al., 2019a). CQA derivatives have been reported as a major compound in the sugarcane top of the fraction possessing potent antioxidant properties (Maeda et al., 2010). Therefore, sugarcane top might be a promising bioresource containing neuroactive chemicals.

In the present study, the senescence-accelerated mouse strain SAMP8 was used as an *in vivo* model (Rhea and Banks, 2017) to evaluate the effect of sugarcane top extract on pathological aging. This non-transgenic strain shows similar neuropathological features of neurodegenerative diseases such as AD, and encompasses Aβ alterations, increased oxidative stress, augmented tau phosphorylation, as well as learning and memory deficits (Butterfield and Poon, 2005; Pallas et al., 2008; Takeda, 2009; Morley et al., 2012). We used human-derived cells to characterize the effect and potential mechanisms of sugarcane top extract on neuronal energy metabolism and neural stem cell fate *in vitro*. Chemical analysis of the extract from sugarcane tops was performed to unveil its composition, and then several *in vivo* and *in vitro* biological assays were performed to examine the possible effect of the extract on cognitive health.

## Materials and Methods

### Preparation of Sugarcane Top Ethanolic Extract (STEE)

Sugarcane (*Saccharum officinarum* L.) tops were collected from a plantation in Asakura, Fukuoka, Japan. Milled sugarcane tops were donated by the Nippo Co., Ltd. (Daito, Osaka, Japan). The milled plant was extracted by maceration with ethanol/water (70:30, v/v) for 2 weeks, with a ratio of plant powder/solvent of 10% (w/v). The extract was filtered through 0.22 μm membranes (Merk Millipore, Billerica, MA, United States) and the dried extract was obtained through rotary evaporation and freeze-drying (yield was approximately 13−14%; 130−140 mg per g of the plant powder).

The extract was dissolved in ethanol/water (70:30, v/v) and stocked for all the *in vitro* experiments. For all the *in vivo* experiments, the extract was dissolved in Mill-Q water.

### Chemical Analysis

The chemical analysis was performed using the Prominence HPLC system (Shimadzu, Kyoto, Japan) equipped with a solvent delivery pump (LC-20AD), autosampler (SIL-20AC), and ELS detector (ELSD-LTII). For the analysis, samples were extracted from milled sugarcane tops using Speed Extractor E-916 (Buchi AG, Uster, Switzerland) with ethanol/water (80:20, v/v). The extracts were evaporated, freeze-dried, and filtered before use. ZORBAX SB-C18 reversed-phase columns (250 × 4.6 mm, 3.5 μm, Agilent, Santa Clara, CA, United States) were used and the column thermostat was maintained at 40°C. The mobile phase consisted of: A. formic acid/water (10:90, v/v), B. acetonitrile/methanol (50:50, v/v) with a 0−100% gradient for 40 min. Chromatography was carried out in gradient mode, using a flow rate of 1.0 mL/min, with detection wavelength at 328 nm. Four concentrations of pure compounds were prepared: 0.2, 0.3, 0.4, and 0.5 μg/mL, as external standards. Each injection volume was 10 μL. Pure chemical compounds of 3-*O*-caffeoylquinic acid (3-CQA), 5-*O*-caffeoylquinic acid (5-CQA) and 3-*O*-feruloylquinic acid (3-FQA) were purchased from the Nagara Science (Gifu, Japan). Isoorientin (ISO, chemically defined as luteolin-6-C-glucoside) was purchased from Sigma-Aldrich (St. Louis, MO, United States). Presented chemical structures were drawn in MarvinSketch (ChemAxon, Budapest, Hungary) software.

### *In vitro* Experiments

#### Cells and Cell Culture

In this study, the human neuroblastoma cell line SH-SY5Y and human fetal neural stem cells (hNSCs) were cultured and used for subsequent experiments *in vitro*. SH-SY5Y cells were purchased from the ATCC (Manassas, VA, United States) and hNSCs (StemPro Neural Stem Cells, A15654) were from the Gibco-Thermo Fisher (Grand Island, NY, United States). SH-SY5Y cells were cultured in Dulbecco’s modified Eagle’s medium (DMEM)/F12 (1:1, v/v) (Gibco) supplemented with 15% fetal bovine serum (FBS, Gibco), 1% non-essential amino acids solution (NEAA, Wako-Fujifilm, Osaka, Japan), and 1% anti-bacterial penicillin/streptomycin. hNSCs were cultured in knockout DMEM/F12 (1:1, v/v) supplemented with 2% StemPro Neural Supplement, 20 ng/mL basic fibroblast growth factor (bFGF), 20 ng/mL epidermal growth factor (EGF), 2 mM Glutamax Supplement (all above reagents were from Gibco), 6 units/mL heparin, and 200 μM ascorbic acid (proliferation medium). The cells were maintained at 37°C in a 95% air/5% CO_2_ humidified incubator.

SH-SY5Y cells expanded as adherent monolayer cultures and were passaged when the culture was confluent (every 3−4 days, up to 8 passages) using cell detachment reagent TrypLE Express (Gibco). hNSCs expanded as free-floating aggregates (Neurospheres) and were passaged when a sphere diameter was ≤150 μm at the maximum (every 9−11 days, up to 3 passages until cells started unexpected differentiation) by dissociation with StemPro Accutase reagent (Gibco). However, the majority of neurospheres were smaller than 150 μm and the cultures had good viability upon passaging.

#### 3-(4,5-Dimethylthiazol-2-yl)-2,5-Diphenyltetrazolium Bromide (MTT) Assay

The MTT assay was used to determine the metabolic activity and cellular viability of SH-SY5Y cells. Briefly, the mitochondrial oxidoreductase activity was assessed using a colorimetric reaction of MTT, and the activity of the enzyme could be converted into cellular viability. The cells (2 × 10^5^ cells/mL) were cultured in 96-well plates/a 96-well plate for 24 h (DMEM/F12 with 15% FBS, 1% NEAA, and 1% penicillin/streptomycin). Then the cells were treated with different concentrations of STEE mixed with serum-reduced Opti-MEM (Gibco) for 72 h. After the treatment, the culture medium was replaced by MTT solution and the cells were incubated for another 6 h to crystallize. Crystalized MTT was dissolved in 10% SDS and the optical density (OD) was measured at 570 nm with a microplate reader Varioskan LUX (Thermo Fisher Scientific, Rockford, IL, United States).

#### Flow Cytometry

Total viable cell numbers were determined using flow cytometry. SH-SY5Y cells were plated in a 6-well plate (2 × 10^5^ cells/mL) and stabilized for 24 h. The cells were then treated with 50 μg/mL of STEE for different time durations: 0, 12, 24, 48, and 72 h. After the treatment, the cells were detached with TrypLE Express and placed in flow cytometry tubes. Cell suspension was diluted in Guava ViaCount Reagent (Luminex, Austin, TX, United States) to immunolabel viable cells. Finally, the total viable cell numbers were determined using ViaCount assay with flow cytometer Guava easyCyte 8HT (Luminex).

#### Measurement of Intracellular ATP Production

Intracellular ATP levels were measured by using a bioluminescence assay kit (Toyo Ink, Tokyo, Japan). The cells were seeded in a 96-well plate at a density of 2 × 10^5^ cells/mL and were incubated for 24 h. Then the cells were treated with 50 μg/mL of STEE for different time durations: 12, 24, and 48 h. After the treatment, the cells were dissolved in luciferin−luciferase solution and the suspensions were transferred to a white bottom 96-well plate. ATP levels were measured as luminescence with a Varioskan LUX multimode multiplate reader.

#### Thymidine Analog Incorporation *in vitro*

The cells were labeled with the synthetic nucleoside 5-Bromo-2’-deoxyuridine (BrdU, Tokyo Chemical Industry, Tokyo, Japan) to identify the proliferation rates of hNSC. The neurospheres were cultured with different concentrations (10, 25, and 50 μg/mL) of STEE for 24 h, and subsequently, 10 μM of BrdU was added to the medium. The cells were incubated for another 24 h; after that, the neurospheres were dissociated with Accutase reagent and plated on Geltrex matrix solution (Gibco) pre-coated culture vessels with knockout DMEM/F12 (1:1, v/v) containing 2% StemPro Neural Supplement, 2 mM Glutamax Supplement, 6 units/mL heparin, and 200 μM ascorbic acid (differentiation medium). After the incubation in the medium containing different concentrations of STEE for 12 h, the cells were processed for immunostaining or RNA extraction.

#### hNSC Differentiation

The cells were cultured as an adherent to let the hNSC differentiate into neural lineages (Schmuck et al., 2017). The neurospheres were pre-treated with different concentrations (10, 25, and 50 μg/mL) of STEE for 24 h and then were dissociated with Accutase reagent. After dissociation, the cells were plated on the Nunc Lab-Tek Chamber Slide System (Thermo Fisher Scientific) pre-coated with Geltrex solution and cultured in differentiation medium containing different concentrations of STEE for 7 days. After the differentiation experiments, each culture was used for subsequent immunocytochemical analysis.

#### Immunocytochemistry

The cells were washed once briefly with PBS, fixed with 4% ice-cold paraformaldehyde (PFA) for 30 min, and then permeabilized with 0.2% Triton-X for 5 min. For BrdU detection, the following DNA denaturation procedure was done−cells were incubated with 2 N HCl for 30 min and 0.1 M borate buffer (0.1 M Na_2_B_4_O_7_, pH = 8.5) for another 15 min. After three washes with PBS, cells were incubated with 5% normal goat serum for 1 h at room temperature to block non-specific binding, and subsequently incubated with the following primary antibodies diluted in blocking solution (1% normal goat serum in PBS) for overnight at 4°C: rabbit monoclonal anti-HuC + HuD (HuC/D, 1:500, Abcam, Cambridge, United Kingdom) and mouse monoclonal anti-BrdU (1:200, Invitrogen, Carlsbad, CA, United States) to detect neural progenitors or thymidine analog; rabbit polyclonal anti-GFAP (1:1000, Novus Biologicals, Centennial, CO, United States) and mouse monoclonal anti-beta III tubulin (Tuj1, 1:1000, Abcam) to detect differentiated astrocytes or neurons. After the incubation and PBS washes, cells were incubated with secondary antibodies diluted in PBS for 1 h at room temperature in the dark. Secondary antibodies conjugated to Alexa Flour 488 or 594 (1:500, Abcam) were used. After the incubation and PBS washes, coverslips were mounted onto glass slides with drops of ProLong Diamond (Invitrogen), including DAPI to stain nuclei. Fluorescent imaging was observed with a confocal microscope TCS SP8 (Leica, Germany).

ImageJ software (National Institutes of Health, Bethesda, MD, United States) was used for counting marker-positive cells, and the length of astrocytic processes were measured using the NeuronJ plugin for ImageJ. Three independent experiments were performed and 8-10 randomly captured sections per well were analyzed.

### *In vivo* Experiments

#### Animals

We used 16-weeks-old male SAMP8 mice (Japan SLC, Shizuoka, Japan) for in vivo experiments. The SAMP8 mice have spatial learning impairments from 12 weeks of age and spatial memory loss commencing from 16 weeks of age (Ikegami et al., 1992; Flood and Morley, 1997; Cheng et al., 2008; Rhea and Banks, 2017). Also, senescence-accelerated mouse resistant 1 (SAMR1) mice, which have a SAM-related genotype and show resistance to accelerated senescence, were used as controls for SAMP8 as described before (Takeda, 2009). The mice were housed individually under controlled temperature (21−23°C) and photoperiod (12-h light/dark) and provided food and water *ad libitum*. All animal procedures were performed according to the guidelines of the Council of Physiological Society, Japan. Experimental protocols were approved by the Ethics Animal Care and Use Committee (18-356), University of Tsukuba, Japan.

#### STEE Administration and Thymidine Analog Incorporation *in vivo*

After 1 week of acclimatization, SAMP8 mice were randomly divided into two groups: SAMP8 water-administered group (*n* = 10) and STEE-administered group (*n* = 10). SAMR1 water-administered group (*n* = 10) were housed as normal aging controls. STEE was dissolved in Mill-Q water and orally administered to SMAP8 mice at 20 mg/kg for 30 days. This concentration was determined following our previous study (Sasaki et al., 2013). SAMP8 controls and SAMR1 mice were administered the equal volume of Mill-Q water to that of the extract. After completion of the 30 days administration, the mice were tested in the behavioral test for 8 days, and the administration was continued during this period also.

BrdU was administered to the mice via the drinking water (Tough and Sprent, 1996; Sasaki et al., 2019a). Briefly, BrdU diluted in drinking water at 1 mg/mL was given to the mice for nine consecutive days starting from the 14th day of STEE administration.

#### Morris Water Maze (MWM)

The MWM test was performed to evaluate the learning and memory ability of mice, according to our previous studies (Sasaki et al., 2013, 2019a). The MWM consisted of a circular pool (120 cm in diameter and 45 cm in height) filled with water (30 cm in depth) kept at 23 ± 2°C. Provisional four quadrants were set in the pool, and an invisible escape platform (10 cm in diameter) was installed and submerged 1 cm below the surface of the water at the midpoint of one quadrant. In the trials, the mice were allowed to swim to escape from the water (to land on the platform) within 60 s. The mice were given four tests each day for 7 days.

On the final day, the escape platform was removed, and the mice were allowed to swim freely for 60 s (the probe test). The number of crossings over the position where the platform was located, and the time spent in the target quadrant were recorded.

### Immunohistochemistry

After 24 h of the MWM probe trial, the mice were sacrificed by cervical dislocation. The brains were extracted from mice for immunohistochemical analysis, fixed in 4% PFA for 14 days at 4°C, then equilibrated in 30% sucrose/PBS (w/v) for 48 h at 4°C. Coronal brain sections (30 μm) were obtained using the SM2010R sliding microtome (Leica). Sections were stored at −20°C in cryoprotectant solution (ethylene glycol, glycerol, 0.1 M phosphate buffer, pH 7.4, 1:1:1:2 by volume) until usage. For immunolabeling, every sixth tissue sections were mounted on SuperFrost Ultra Plus Adhesion slides (Thermo Fisher Scientific) and allowed to dry for at least 30 min at room temperature. Slides were washed in PBS to remove excess cryoprotectant solution. Antigen retrieval was performed for immunolabeling with anti-BrdU antibody by heating slides incubated in 2N HCl solution for 20 min at 42°C. Slides were rinsed in PBS three times before permeabilization for 15 min in PBS containing 0.03% Triton X-100 (PBS-T). Sections were blocked in 10% donkey serum in PBS-T for 1 h at room temperature. Primary antibodies were diluted, as specified below, in blocking buffer and incubated overnight. Sections were washed then incubated with fluorochrome-conjugated specific secondary antibodies for 2 h at room temperature. Slides were coverslipped using FluorSave reagent (Merck Millipore).

For evaluation of results, images were obtained using a Nikon Ti-Eclipse microscope (Nikon, Japan). For quantification, confocal images were obtained with a Zeiss LSM 710 laser scanning confocal microscope (Leica) with 1 μM Z-stacks for stereological quantification. Primary antibodies used were: rat monoclonal anti-BrdU (1:200, Abcam), rabbit polyclonal anti-DCX (1:200, CST, Danvers, MA, United States), and chicken polyclonal anti-GFAP (1:200, Abcam). Every eighth tissue section was sampled for quantification. Using tiled images, the whole dentate gyrus was quantified using ImageJ software in a blinded fashion. At least three sections were counted per animal.

### ELISA

The cerebral cortex was isolated from the extracted brain and homogenized with ultrasonication in RIPA Lysis Buffer (Santa Cruz, Dallas, TX, United States), including protease inhibitors, on ice. The homogenates were centrifuged at 16,000 *g* for 20 min, and the resulting supernatant was used for the measurement of neurotransmitters by ELISA.

The monoamine levels (dopamine; DA, norepinephrine; NE, and serotonin; 5-HT) in the tissue were determined with a sandwich ELISA (ImmuSmol, Talence, France). Acetylcholine (ACh) levels were measured by the ELISA kit (BioVison, Milpitas, CA, United States) according to the manufacturer’s instructions. A 2D Quant kit (GE Healthcare, Piscataway, NJ, United States) was used to normalize each neurotransmitter level to total protein concentration.

### Transcriptome Analysis

#### RNA Isolation

Total RNA was isolated from SH-SY5Y cells or neurospheres treated with STEE for 24 h, adherent hNSCs dissociated from STEE pre-treated neurospheres, and cerebral cortices of the mice. ISOGEN kit (Nippon Gene, Toyama, Japan) was used to extract total RNA from cells according to a protocol as previously described (Han et al., 2010).

#### Reverse Transcription and qRT-PCR

Isolated RNAs were reverse transcribed with SuperScript IV VILO Master Mix (Applied Biosystems, Foster City, CA, United States) according to the manufacturer’s protocol.

Quantitative real-time polymerase chain reaction (qRT-PCR) was performed on Applied Biosystems 7500 RT-PCR System as follows: 2 min at 50°C, 10 min at 95°C and then 45 cycles of PCR (15 s at 95°C; 1 min at 60°C). The primers (Applied Biosystems) used for RT-PCR were: *ACTB* (Hs01060665_g1), *GAPDH* (Hs02786624_g1), *PGK1* (Hs00943178_g1), *PGAM1* (Hs01652468_g1), *PKM* (Hs00761782_s1), *PC* (Hs01085875_g1), *TUBB3* (Hs00801390_s1), *GFAP* (Hs00909233_m1), *PDGFRA* (Hs00998018_m1), *NES* (Hs04187831_g1), *ASCL1* (Hs00269932_m1), and *HES1* (Hs00172878_m1). Amplification was performed with TaqMan Gene Expression Master Mix (Applied Biosystems). *ACTB* or *GAPDH* levels were used for internal controls, and the relative expression levels of each transcript were determined in triplicate from three independent experiments.

#### Microarray Gene Expression Profiling

RNA samples extracted from cerebral cortices were amplified and biotinylated with Affymetrix 3’ IVT PLUS Reagent Kit (Affymetrix, Santa Clara, CA, United States), according to the manufacturer’s instructions. Biotinylated complementary RNA (cRNA) was hybridized onto Affymetrix Mouse Genome 430 PM array strips (Affymetrix) for 16 h at 45°C in the hybridization station. The hybridized arrays were washed, stained, and scanned with GeneAtlas Fluidics and Imaging Station. Then the gene-level information (CHP files) was obtained from the probe intensity files (CEL files) using robust multichip analysis (RMA) summarization algorithm in Expression Console software (Affymetrix^1^). Subsequent analysis of the obtained data was carried out in Transcriptome Analysis Console (TAC) ver.4 software (Thermo Fisher Scientific). Microarray was conducted for two mRNA biological samples of each group. Differentially expressed genes (DEGs) with *p*-Value < 0.05 (one-way between-subjects ANOVA) were included for gene ontology (GO) analysis. The Molecular Signatures Database (MSigDB) of Gene Set Enrichment Analysis (GSEA) web tool was used to determine whether the gene sets show statistically significant and concordant differences between two biological states^2^ (Subramanian et al., 2005; Liberzon, 2014). Graphical presentations were generated in Microsoft Excel 2016 (Microsoft, Redmond, WA, United States). Heatmaps were generated using Morpheus software^3^. Venn-diagram was generated using an open source tool^4^. Microarray dataset are deposited in the Gene Expression Omnibus (GEO) under Accession Number: GSE151727^5^.

### Statistical Analysis

Data are presented as means ± standard error of means (SEM) unless otherwise indicated. Normal distribution was tested by the Shapiro−Wilk normality test. Student’s *t*-tests (unpaired) were performed to determine significant difference between two groups. A one-way analysis of variance (ANOVA) followed by Dunnett’s *post hoc* test was applied to compare each of the treatment conditions with a single control group. Differences in viable cell numbers between the treatment groups at various time points were assessed using two-way ANOVA followed by Sidak’s *post hoc* test. For *in vivo* experiments, differences in escape latency among the treatment groups at different time points were assessed using two-way repeated-measures ANOVA with Fisher’s LSD test. Other behavioral tests were compared using either one-or two-way (for repeated measures) ANOVA with Fisher’s LSD test for normally distributed data. In the case of non-normally distributed data, the Kruskal−Wallis test followed by Dunn’s *post hoc* test was performed. The level of significance was set at α < 0.05. All the statistical analyses and graphical representations were performed using GraphPad Prism 8 (GraphPad, San Diego, CA, United States).

## Results

Firstly, we evaluated the effect of STEE on age-associated decline in spatial learning and memory in SAMP8 mice using the MWM test. Secondly, we determined the effects of STEE on hippocampal neurogenesis and cortical monoamine levels in SAMP8 mice brains by immunohistochemical and biochemical analysis, respectively. Next, we performed a comprehensive transcriptome analysis of the cortex by microarray to reveal beneficial biological events regulated by STEE, which were further validated in a variety of experiments in human fetal brainderived neurospheres and in human SH-SY5Y cells.

### HPLC Revealed Major Polyphenolic Constituents in the Sugarcane Top

**FIGURE 1:**
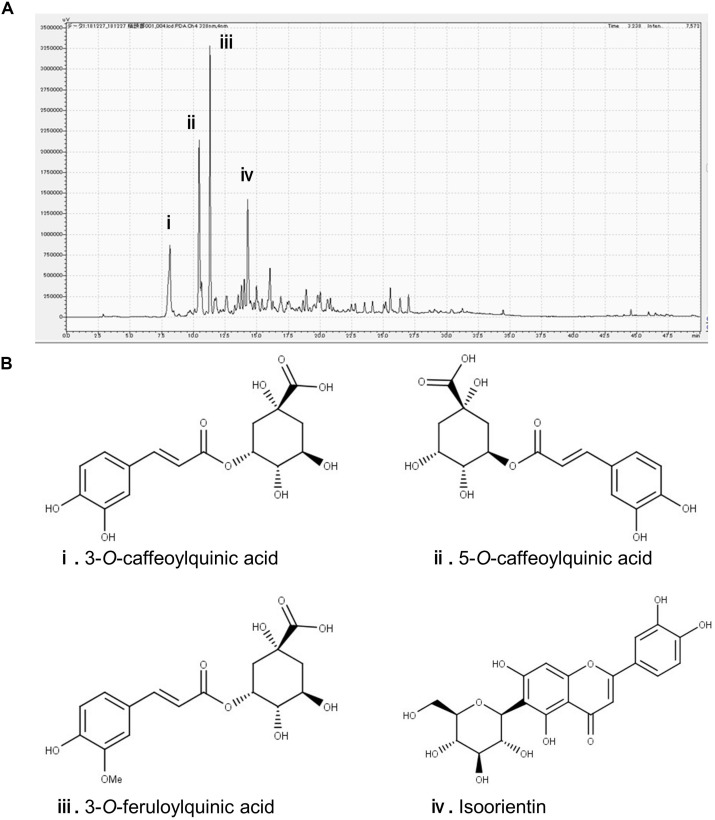
Identification of the major polyphenolic constituents of sugarcane top extract through HPLC. The gradient profile was 0–100%, 40 min and the detection wavelength was at 328 nm. **(A)** Representative HPLC chromatogram of the extract. **(B)** Major peaks were identified as 3-*O*-caffeoylquinic acid **(i)**, 5-*O*-caffeoylquinic acid **(ii)**, 3-*O*-feruloylquinic acid **(iii)**, and isoorientin **(iv)**. The concentrations of the phenolic compounds in the extract were measured at the peaks as **(i)** 3.52 ± 0.15, **(ii)** 4.91 ± 0.06, **(iii)** 6.24 ± 0.51, and **(iv)** 4.27 ± 0.01 mg/g.

In the chromatogram generated by HPLC, we observed four major peaks (Figure 1A). Peaks were identified as derived from 3-CQA, 5-CQA, 3-FQA, and ISO, respectively (Figure 1B). The concentrations of 3-CQA, 5-CQA, 3-FQA and ISO in the extract were determined as approximately 3.52 ± 0.15, 4.91 ± 0.06, 6.24 ± 0.51, and 4.27 ± 0.01 mg, respectively (per g of the extract).

### STEE Ameliorated Spatial Learning and Memory Deficit in SAMP8 Mice

**FIGURE 2:**
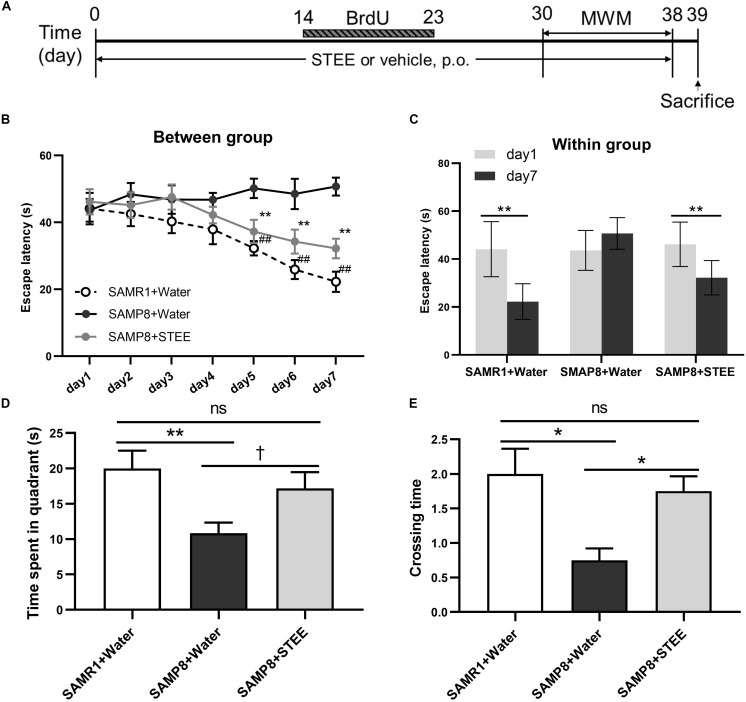
Effects of STEE on spatial learning and memory determined with the Morris Water Maze. **(A)** Experimental timeline. **(B)** Escape latency of each treatment group at different time points. Asterisk refers to significant difference between water and STEE-fed SAMP8 mice (SAMP8 + Water vs. SAMP8 + STEE; **p < 0.01). Hash sign refers to significant difference between SAMP8 and SAMR1 control mice given water (SAMP8 + Water vs. SAMR1 + Water; ^##^*p* < 0.01). The differences among treatment groups were assessed using two-way repeated-measures ANOVA followed by Fisher’s LSD test. **(C)** Escape latency of each treatment group before and after 7-day training sessions in MWM tests: **p < 0.01. **(D)** Effects of STEE on time spent in target quadrant in the probe test. Differences between the groups were analyzed using one-way ANOVA with Fisher’s LSD test: **p < 0.01, †*p* = 0.054; ns, non-significant. **(E)** Effects of STEE on numbers of crossing over the platform in the probe test. Pair-wise comparisons among the groups were performed using Kruskal-Wallis test followed by Dunn’s *post hoc* test: *p < 0.05; ns, non-significant. Values are presented as mean ± *SEM* (*n* = 6 animals per group).

SAMP8 mice were administered STEE extract (20 mg/kg, p.o.) or water for 30 days, then the mice were tested in the MWM to evaluate their memory (experimental timeline is shown in Figure 2A). In the MWM test, the animals were trained to find the hidden platform for 7 days, with 4 trials per day. The time the mice spent to find the platform was analyzed and termed as escape latency. In the training sessions, the escape latency of the STEE-fed mice was significantly reduced in comparison with the SAMP8 controls from the 5th to the 7th day of the test (Figure 2B). Also, escape latency in the STEE-fed mice significantly decreased on days 5, 6, and 7 of the trial compared to day 1 (before the trial). There was no significant difference in escape latency before and after the trial sessions in the SAMP8 controls (Figure 2C). However, the SAMR1+water and SAMP8+STEE groups both showed significantly reduced escape latency between day 1 and day 7.

The time spent in the target quadrant tended to increase in the STEE-fed mice (*p* = 0.054) compared to the SAMP8 controls (Figure 2D). In the probe test (on the 8th day), there was a significant difference in the crossing time across the virtual platform between the STEE-fed mice and the SAMP8 controls (Figure 2E). No difference was observed in the behavioral tests between SAMP8+STEE and SAMR1 control.

Taken together, these data demonstrate the effect of STEE on the recovery of cognitive functions in SAMP8 mice.

### STEE Tended to Induce Adult Hippocampal Neurogenesis (AHN) of SAMP8 Mice

**FIGURE 3:**
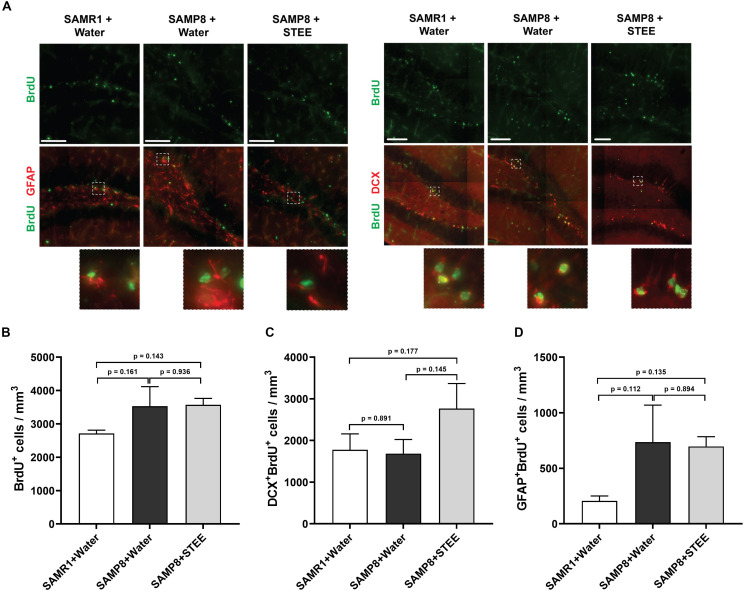
Levels of proliferation and differentiation markers in the hippocampal SGZ. **(A)** Representative images of immunolabeling for BrdU, DCX, and GFAP in SAMR1 mice given water, SAMP8 mice given water and SAMP8 mice given STEE. Scale bar: 100 μm. **(B)** Total number of BrdU positive proliferating cells in the SGZ of animals. **(C)** Number of DCX/BrdU double-positive newborn immature neurons. **(D)** Number of GFAP/BrdU double-positive cycling astrocytic stem cells and progenitors. The error bar represents the ± *SEM* (*n* = 3 animals per group). Comparisons were performed through one-way ANOVA with Fisher’s LSD test.

We examined the subgranular zone (SGZ) because the MWM is a hippocampal-dependent task, while spatial memory is strongly associated with SGZ neurogenesis (Anacker and Hen, 2017). All animals were given BrdU in their drinking water to label dividing cells during treatment and BrdU labeling in the SGZ was quantified. Immunolabeling for BrdU revealed no significant changes between groups [*F*(2, 6) = 1.798, *p* = 0.244] (Figures 3A,B), indicating no statistically significant difference in neural proliferation in the SGZ between SAMR1 mice and SAMP8 controls given water, as well as between SAMP8 controls given water and SAMP8 mice given STEE. We then investigated whether neurogenesis might be affected. We, therefore, quantified the proportion of newborn neurons (BrdU^+^DCX^+^) (Figure 3C) or progenitors and cycling astrocytic stem cells (BrdU^+^GFAP^+^) (Figure 3D). As a result, no significant difference was found between groups, although STEE showed a non-significant trend of elevated BrdU^+^DCX^+^ newborn neurons (approximately 1.6-fold increase).

These results indicate that the cognitive decline of SMAP8 was independent of AHN, but that the rescue effect of STEE might be correlated with the increased AHN and further neurodevelopment.

### STEE Restored Levels of Neurotransmitters in SAMP8 Brains

**FIGURE 4:**
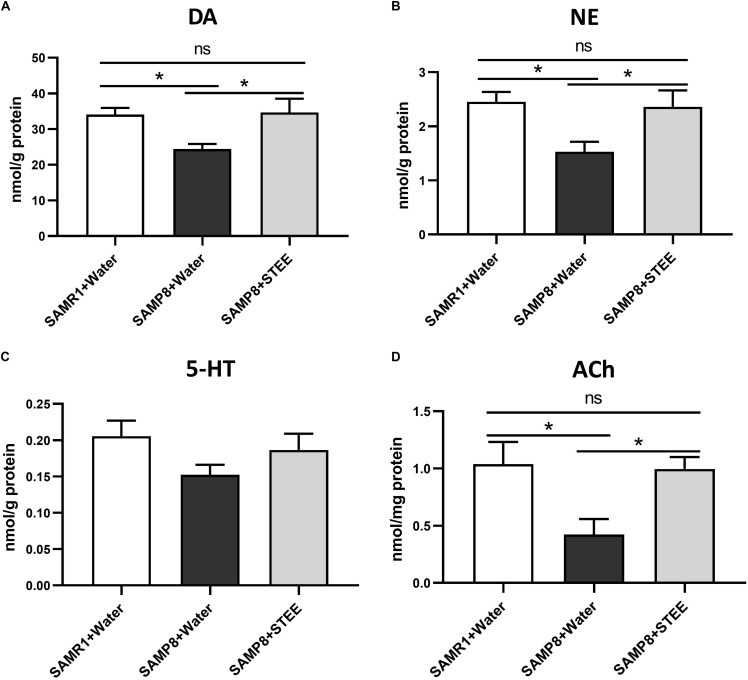
Levels of neurotransmitters in cerebral cortices. DA **(A)**, NE **(B)**, 5-HT **(C)**, and ACh **(D)** levels in brain tissues were measured by ELISA. Values are presented as mean ± *SEM* (n = 5 animals per group). Comparisons were performed using one-way ANOVA with Fisher’s LSD test: *p < 0.05; ns, non-significant.

The cerebral cortex, which plays a critical role in cognition and memory, was separated and collected from the extracted brains of mice. Neurotransmitter levels in the cortical tissue homogenates were measured by ELISA. DA, NE, and ACh levels in the cerebral cortices were significantly reduced in SAMP8 controls compared to SAMR1 mice, whereas oral administration of STEE restored neurotransmitter levels in the cortices of SAMP8 mice (Figures 4A,B,D). There was a slight decrease in 5-HT levels (*p* = 0.078) in the cortices of SAMP8 compared to SAMR1 mice. This tendency was partially reversed by STEE administration (*p* = 0.262) (Figure 4C).

The above findings indicate that increased neurotransmitter levels in the cerebral cortex might be correlated with the rescue of age-related memory loss in SAMP8 mice.

### STEE Regulated a Wide Range of Biological Processes in the Cerebral Cortex of SAMP8 Mice

**FIGURE 5:**
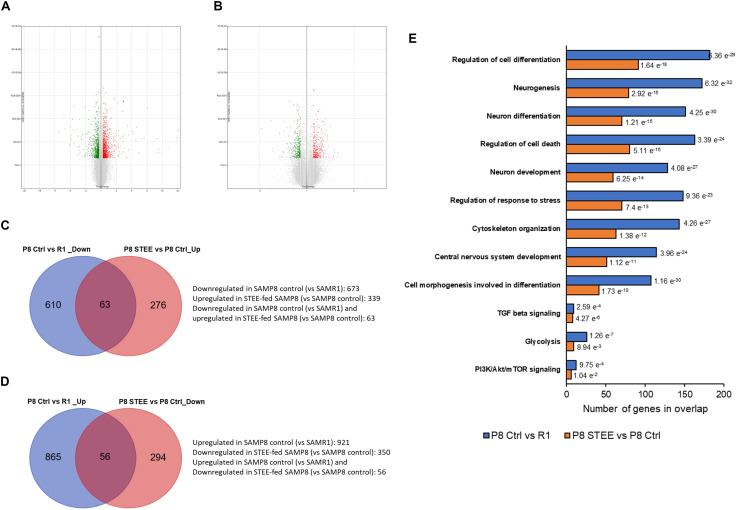
Volcano plot displaying DEGs between **(A)** SAMP8 control and SAMR1, and **(B)** STEE-fed SAMP8 and SAMP8 control. In the volcano plot, the vertical axis (*y*-axis) corresponds to –log10 *p*-Value of the ANOVA *p*-Values, and the horizontal axis (*x*-axis) displays linear fold change. The red dots represent the upregulated genes; the green dots represent the downregulated genes. Venn diagram showing common and unique sets of DEGs between the groups. **(C)** Blue circle denotes downregulated DEGs in SAMP8 control compared to SAMR1, red circle denotes upregulated DEGs in STEE-fed SAMP8 compared to SAMP8 control. **(D)** Blue circle denotes upregulated DEGs in SAMP8 control compared to SAMR1, red circle denotes downregulated DEGs in STEE-fed SAMP8 compared to SAMP8 control. **(E)** Significantly enriched biological processes by the DEGs between the groups (*p* < 0.05; modified Fisher’s exact test). Each bar represents the number of DEGs in overlap. P values for each gene set are shown at the outside end of each bar.

Microarray analysis was performed on cerebral cortex of the mice to investigate the mechanism underlying the effects of STEE. Volcano plots in Figures 5A,B show the significantly regulated genes (SAMP8 control vs. SAMR1 mice and STEE-fed SAMP8 vs. SAMP8 control, respectively). After data processing, we identified 1594 unique DEGs (921 upregulated, 673 downregulated) in SAMP8 controls compared to SAMR1 mice, and 689 unique DEGs (339 upregulated, 350 downregulated) in STEE-fed SAMP8 compared to SAMP8 controls. Distribution of fold changes of DEGs is shown in Supplementary Figure 1.

Venn diagrams show common and unique sets of DEGs between the groups (Figures 5C,D). Sixty-three genes were downregulated in SAMP8 control (vs. SAMR1) but were upregulated in STEE-fed SAMP8 (vs. SAMP8 control). And, 56 genes were upregulated in SAMP8 control (vs. SAMR1) but downregulated in STEE-fed SAMP8 (vs. SAMP8 control). Among these 119 genes which were regulated in the same direction both in STEE-fed SAMP8 and SAMR1 (compared to SAMP8 control), four genes are associated with transforming growth factor (TGF) beta signaling (systemic name: M5896); nine genes are associated with protein kinase activity (GO:0004672); eight genes are associated with presynapse (GO:0098793); 13 genes are associated with neuron projection (GO:0043005).

GSEA revealed significantly enriched gene sets by the DEGs between SAMP8 control and SAMR1 mice, and between STEE-fed SAMP8 and SAMP8 control. Top significantly enriched biological processes include, but not limited to, regulation of cell differentiation (GO:0045595), neurogenesis (GO:0022008), neuron differentiation (GO:0030182), regulation of cell death (GO:0010941), neuron development (GO:0048666), regulation of response to stress (GO:0080134), cytoskeleton organization (GO:0007010), central nervous system development (GO:0007417), and cell morphogenesis involved in differentiation (GO:0000904). Also, Hallmark gene sets TGF beta signaling, glycolysis (systemic name: M5937), and phosphoinositide 3-kinase (PI3K)/protein kinase B (Akt)/mammalian target of rapamycin (mTOR) signaling (systemic name: M5923) were enriched by the DEGs. Hallmark gene sets summarize and represent specific well-defined biological states or processes and display coherent expression (Figure 5E). Biological processes are arranged in the bar chart according to *p*-Value (hypergeometric *p*-Value).

### STEE Regulated Expression of Genes Associated With Neurotrophin Signaling, Glucose Metabolism, and Neural Development in Cerebral Cortex of SAMP8 Mice

**FIGURE 6:**
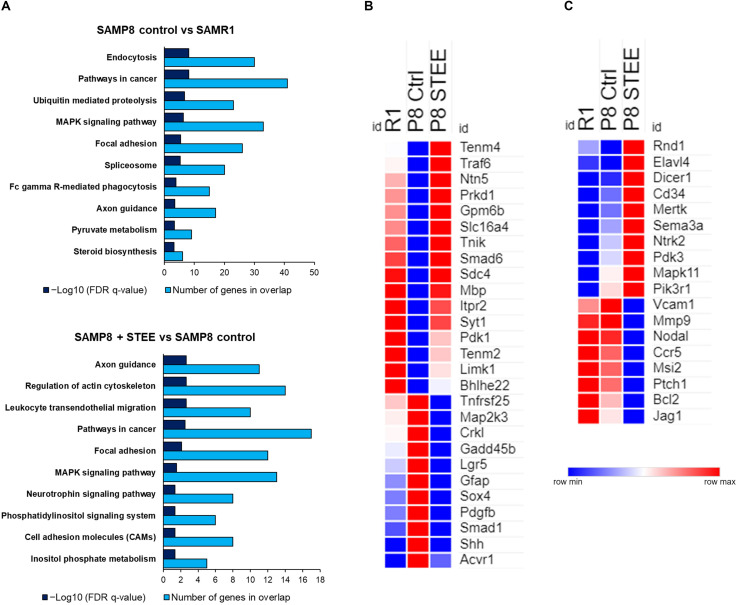
Functional analysis of DEGs between the groups. **(A)** Top 10 significantly enriched KEGG pathways by the DEGs between the groups (*p* < 0.05; modified Fisher’s exact test). Heatmaps showing relative expression intensity of genes. **(B)** Genes regulated in the same direction both in SAMR1 controls and STEE-fed SAMP8 compared to SAMP8 controls. **(C)** Genes regulated specifically in SAMP8 fed with STEE. Represented genes in heatmaps are mainly involved in neurotrophin signaling, glucose metabolism, and neurodevelopment.

Figure 6A shows the top 10 significantly enriched Kyoto Encyclopedia of Genes and Genomes (KEGG) pathways by the DEGs between the groups according to false discovery rate (FDR) *q*-Value. DEGs between SAMP8 control and SAMR1 mice significantly enriched endocytosis, cancer, ubiquitin-mediated proteolysis, and mitogen-activated protein kinase (MAPK) signaling pathways as well as axon guidance and pyruvate metabolism pathways. On the other hand, axon guidance and regulation of cytoskeleton are the top-ranked KEGG pathways enriched by the DEGs between STEE-fed SAMP8 and SAMP8 control. Neurotrophin signaling, phosphatidylinositol signaling system, and cell adhesion molecules (CAMs) are also included among the top 10 enriched pathways between STEE-fed SAMP8 and SAMP8 control.

The heatmaps show the relative intensity of the genes which were regulated in the same direction (Figure 6B) both in STEE-fed SAMP8 and SAMR1 compared to SAMP8 control, or which were regulated specifically in STEE-treated SAMP8 (Figure 6C). The relative intensity is shown as average of duplicates for each. Presented genes are mainly involved in neurotrophin signaling, glucose metabolism, and neural development. Heatmap in Figure 6B shows the average expression intensities of Teneurin transmembrane protein 4 (*Tenm4*), TNF receptor-associated factor (Traf) 6 (*Traf6*), Netrin 5 (*Ntn5*), Protein kinase D1 (*Prkd1*), Glycoprotein m6b (*Gpm6b*), Solute carrier family 16 (monocarboxylic acid transporters), member 4 (*Slc16a4*), TRAF2 and NCK interacting kinase (*Tnik*), SMAD family member 6 (*Smad6*), Syndecan 4 (*Sdc4*), Myelin basic protein (*Mbp*), Inositol 1,4,5-trisphosphate receptor type 2 (*Itpr2*), Synaptotagmin I (*Syt1*), Pyruvate dehydrogenase kinase, isoenzyme 1 (*Pdk1*), Teneurin transmembrane protein 2 (*Tenm2*), LIM-domain containing, protein kinase (*Limk1*), Basic helix-loop-helix family, member e22 (*Bhlhe22*), Tumor necrosis factor receptor superfamily, member 25 (*Tnfrsf25*), Mitogen-activated protein kinase kinase 3 (*Map2k3*), V-crk sarcoma virus CT10 oncogene homolog (avian)-like (*Crkl*), Growth arrest and DNA-damage-inducible 45 beta (*Gadd45b*), Leucine rich repeat containing G protein coupled receptor 5 (*Lgr5*), Glial fibrillary acidic protein (*Gfap*), SRY-box containing gene 4 (*Sox4*), Platelet derived growth factor, B polypeptide (*Pdgfb*), SMAD family member 1 (*Smad1*), Sonic hedgehog (*Shh*), and Activin A receptor, type 1 (*Acvr1*). Heatmap in Figure 6C shows the average expression intensities of Rho family GTPase 1 (*Rnd1*), ELAV (embryonic lethal, abnormal vision, Drosophila)-like 4 (Hu antigen D) (*Elavl4*), Dicer 1, ribonuclease type III (*Dicer1*), CD34 antigen (*Cd34*), C-mer proto-oncogene tyrosine kinase (*Mertk*), Sema domain, immunoglobulin domain (Ig), short basic domain, secreted, (semaphorin) 3A (*Sema3a*), Neurotrophic tyrosine kinase, receptor, type 2 (*Ntrk2*), Pyruvate dehydrogenase kinase, isoenzyme 3 (*Pdk3*), Mitogen-activated protein kinase 11 (*Mapk11*), Phosphatidylinositol 3-kinase, regulatory subunit, polypeptide 1 (p85 alpha) (*Pik3r1*), Vascular cell adhesion molecule 1 (*Vcam1*), Matrix metallopeptidase 9 (*Mmp9*), Nodal (*Nodal*), Chemokine (C-C motif) receptor 5 (*Ccr5*), Musashi RNA-binding protein 2 (Msi2), Patched homolog 1 (*Ptch1*), B cell leukemia/lymphoma 2 (*Bcl2*), and Jagged 1 (*Jag1*). Characteristics of each gene presented in the heatmaps are shown in the Supplementary Tables 1, 2. A schematic diagram has been presented showing the possible mechanism of action and future research scope of STEE (Supplementary Figure 2).

### STEE Enhanced Cellular Energy Metabolism Through Upregulation of Glycolytic Reaction in SH-SY5Y Cells

The MTT assay was conducted in SH-SY5Y cells treated with different doses of STEE to evaluate its effect on metabolic activity and cellular viability. STEE treatment increased SH-SY5Y cell MTT activity (129% at the maximum) at a dose of 10 μg/mL and above. On the other hand, no significant difference was observed after STEE treatment in the numbers of total viable cells between groups at all time points (12, 24, 48, and 72 h). These data indicate that STEE does not affect neuronal cell viability or proliferation but may affect cell metabolic activity. The data is shown in Supplementary Figure 3.

**FIGURE 7:**
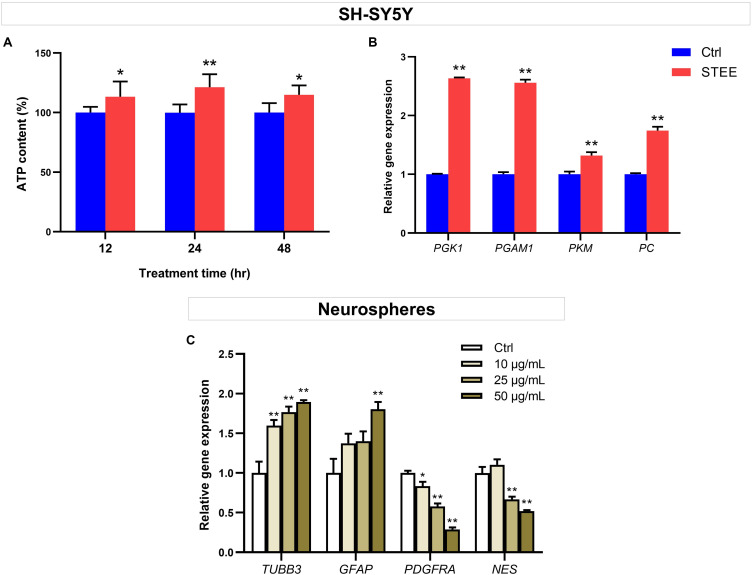
Effect of STEE on neuronal energy metabolism and neurodevelopmental regulation. **(A)** Intracellular ATP content in SH-SY5Y cells at different time points (12, 24, and 48 h after treatment) were measured as emission intensity. The cells were treated with 50 μg/mL of STEE (or non-treatment). Results are expressed as relative to control percentages and presented as mean ± *SD*. Comparisons between two groups were performed by Student’s *t*-test: *p < 0.05 and **p < 0.01. **(B)** qRT-PCR analyses of *PGK1*, *PGAM1*, *PKM*, and *PC* transcript levels in STEE (50 μg/mL) treated neuronal cells. Results are shown as relative to control values. Values are expressed as mean ± *SEM* (triplicates from three independent experiments). Comparisons were performed using Student’s *t*-test: **p < 0.01. **(C)** Effects of STEE on the expressions of neurodevelopmental genes in neurospheres. *TUBB3* (neuronal marker), *GFAP* (astrocytic marker), *PDGFRA* (oligodendrocytic marker), and *NES* (stem cell marker) transcript levels were determined with qRT-PCR. Results are expressed as relative to control values. Values are presented as mean ± *SEM* (triplicates from three independent experiments). Comparisons were performed using one-way ANOVA followed by Dunnett’s *post hoc* test: *p < 0.05 and **p < 0.01.

To further verify whether enhanced cellular energy metabolism contributed to the above results, intracellular ATP levels were evaluated. ATP is one of the important indicators of cellular energy production. As shown in Figure 7A, 50 μg/mL of STEE increased intracellular ATP levels (121% at the maximum) at all time points (12, 24, and 48 h). These data support that STEE enhanced energy metabolism in neuronal cells.

To corroborate the interaction between promoted ATP levels and energy metabolism, the expressions of several genes involved in glycolytic reactions were evaluated by qRT-PCR. STEE-treated cultures (24 h) exhibited significant increases in Phosphoglycerate kinase 1 (*PGK1*), Phosphoglycerate mutase 1 (*PGAM1*), Pyruvate kinase (*PKM*), and Pyruvate carboxylase (*PC*) transcript levels (Fold change 2.63, 2.55, 1.31, and 1.74, respectively, Figure 7B).

Given the relationship between intracellular ATP production and the glycolytic pathway, these results indicate that STEE stimulated glycolysis and anaplerosis, followed by an increase in cellular energy metabolism.

### STEE Regulated the Expression of Neural Development-Related Genes in Neurospheres

Given the *in vivo* effects of STEE on SGZ, we hypothesized that STEE might enhance neural differentiation. The transcript levels of several neurodevelopmental factors were evaluated in neurospheres to investigate whether STEE affects neural stem cell development. Tubulin beta 3 (*TUBB3*), Glial fibrillary acidic protein (*GFAP*), Platelet-derived growth factor receptor alpha (*PDGFRA*), and Nestin (*NES*) were selected as neuronal, astrocytic, oligodendrocytic, and stem cell markers, respectively. As shown in Figure 7C, 24 h STEE treatment increased the expression of *TUBB3* and *GFAP* in a dose-dependent manner (Fold change 1.89 and 1.80 at the maximum, respectively), whereas the expressions of *PGDFRA* and *NES* were decreased (Fold change −1.71 and −1.47 at the maximum, respectively). These findings suggest that STEE may induce the cells to lose their stem cell features, transform into the transitional type, and differentiate into neurons or astrocytes.

### STEE Affected Proliferation and the Early Phase of Differentiation in hNSCs

**FIGURE 8:**
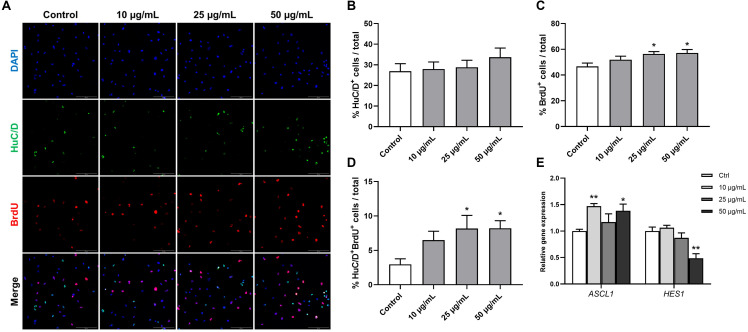
Effects of STEE on proliferation and early differentiation of hNSC. Neurospheres were cultured in proliferation medium with BrdU for 24 h, and BrdU-labeled dissociated cells were visualized by confocal microscopy. **(A)** Fluorescence images of immunocytochemistry for the neuronal progenitor marker HuC/D and the proliferation marker BrdU in control or STEE-treated cells. Scale bar = 100 μm. **(B–D)** Quantification of HuC/D, BrdU, or HuC/D/BrdU double-positive cells over total cells visualized with DAPI. *n* = 8–10 sections per culture. **(E)** Transcript levels of *ASCL1* and *HES1* in the cultures at the early differentiation stage. Transcript levels were determined in triplicates from three independent experiments. **(B–E)** Results are expressed as relative percentages or relative to control values. Values are presented as mean ± *SEM*. Comparisons were performed using one-way ANOVA followed by Dunnett’s *post hoc* test: *p < 0.05 and **p < 0.01.

Human fetal brain derived-neurospheres were exposed to BrdU to assess the effect of STEE on hNSCs proliferation. Cells that went through the S phase of the cell cycle incorporated BrdU, which was detected with immunocytochemistry and co-stained with neural progenitor markers. While several neural progenitor markers have been established, Hu proteins (HuB, HuC, and HuD) are reportedly expressed at very early stages of neuronal development, and continue to be expressed in immature neurons (Akamatsu et al., 1999, 2005). Here, as well as BrdU, HuC/D protein expression was evaluated in the cultures at an early differentiation stage (12 h after plating) to assess the pro-neurogenic effects of STEE (Figure 8A). As shown in Figure 8B, STEE treatment significantly increased the percentage of BrdU^+^ cells (120% at 25 μg/mL STEE-treated group and 122% at 50 μg/mL STEE-treated group) compared to controls. Cultures in the presence of STEE exhibited no changes in the percentages of HuC/D^+^ cells; however, a significant increase was observed in the proportion of HuC/D^+^BrdU^+^ cells (275% at 25 μg/mL STEE-treated group and 276% at 50 μg/mL STEE-treated group) in the cultures treated with STEE compared to untreated control (Figures 8C,D). These results indicate that STEE induces the proliferation of hNSC and activates newly divided cells to differentiate into neuronal cells *in vitro* cultures.

On the basis of these results showing the pro-neurogenic effect of STEE, qRT-PCR analysis was carried out to corroborate the involvement of transcription factors. RNA was extracted from cells cultured under the same conditions as above, and the expression of Achaete-scute homolog 1 (*ASCL1*) and Hairy and enhancer of split 1 (*HES1*) were evaluated. Ascl1 and Hes1 are basic-helix-loop-helix (bHLH) transcription factors. Gene expression dynamics of these factors regulate the quiescence versus activation of neural stem cells in the early developmental phase (Sueda et al., 2019). As expected, *ASCL1* expression showed a significant increase (1.47-fold with 10 μg/mL and 1.38-fold with 50 μg/mL) and *HES1* expression showed a significant decrease (−1.51-fold with 50 μg/mL) in the STEE-treated cultures compared to control (Figure 8E). These findings reflect the stimulation of Ascl1 and Hes1 expression dynamics toward an activation of stem cells.

These results suggest that STEE promoted hNSC proliferation and differentiation at early culture stages due to the dynamic regulation of transcription factors.

### STEE Induced Neural Differentiation and Expansion in hNSCs

**FIGURE 9:**
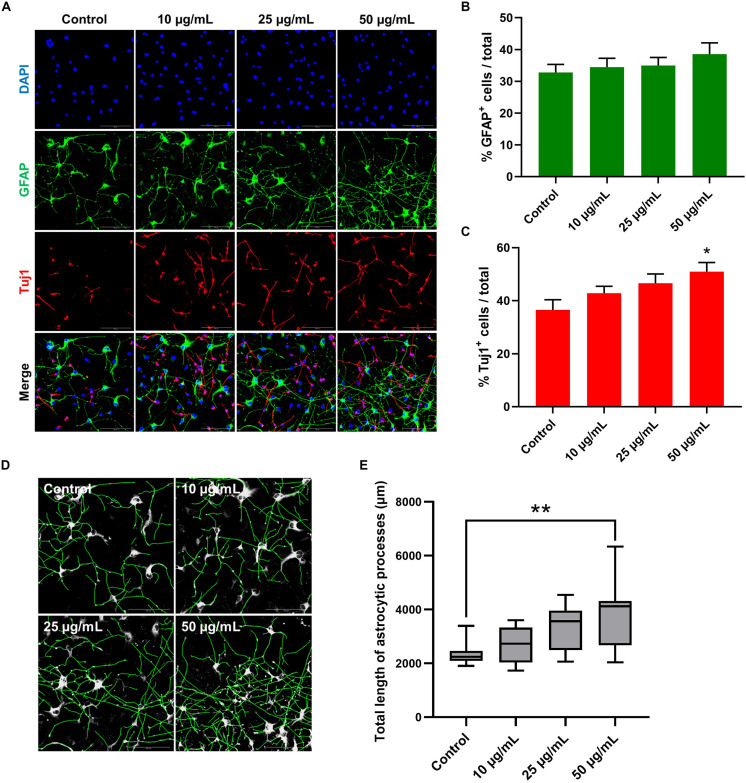
Effects of STEE on hNSC differentiation. hNSCs were cultured as a monolayer for 7 days in differentiation medium only (control cells) or in differentiation medium with different concentrations of STEE (treatment cells). Immunostained cells were visualized by confocal microscopy. **(A)** Fluorescence images of immunocytochemistry for astrocytic marker GFAP and neuronal marker Tuj1 in the control and STEE-treated cells. Scale bar = 100 μm. **(B,C)** Quantification of GFAP or Tuj1 positive cells over total cells visualized with DAPI. Results are expressed as relative percentages. Comparisons were performed using one-way ANOVA followed by Dunnett’s *post hoc* test: *p < 0.05. Error bars represent the ± *SEM* (*n* = 8–10 sections per culture). **(D)** Effects of STEE on the length of astrocytic processes. Representative images of outlined GFAP positive processes, and **(E)** quantification of total length of the processes. Results are shown as box plots. Each box ranges from 25th to 75th percentile, the line in the middle represents the median value, the error bar represents the ± *SEM* (*n* = 8–10 sections per culture). Asterisks refer to statistical significance by Kruskal–Wallis test followed by Dunn’s *post hoc* test: **p < 0.01.

To further investigate the possible effect of STEE on hNSC differentiation, changes in the proportion of cell populations were analyzed in 7-day cultures (Figure 9A). As shown in Figure 9C, STEE exposure increased the percentage of Tuj1^+^ neuronal cells (139% at the maximum). No significant change was observed in the proportion of GFAP^+^ astrocytic cells (Figure 9B); however, their processes were observed to be expanded in STEE-treated cultures (Figures 9A,D). Interestingly, the tracing analysis using ImageJ showed an extended total length of astrocytic processes (approximately 1.6-fold increase at the maximum) (Figure 9E), indicating STEE did not induce differentiation into astrocytes but affected to morphological expansion.

Altogether, these findings suggest that STEE could contribute to neuronal differentiation and astrocyte morphogenesis in *in vitro* cultures.

## Discussion

The present study revealed the polyphenolic constituents of STEE, and demonstrated that they could enhance neuronal energy metabolism and induce neural stem cell development. Moreover, STEE reversed spatial memory deficits in SAMP8 mice.

Several animal models that mimic the symptoms of age-related neurodegenerative diseases, such as AD, have been established. SAMP8 mice precociously and progressively develop a multisystemic aging phenotype, including learning and memory deficits, as well as pathological features similar to those of AD (Butterfield and Poon, 2005; Pallas et al., 2008; Takeda, 2009; Morley et al., 2012). Therefore, SAMP8 mice are considered advantageous compared to transgenic strains for sporadic AD research (Pallas et al., 2008; Ito, 2013). In the present study, we found that treatment of SAMP8 mice with STEE for 30 days significantly improved performance in the MWM behavioral test, indicating that STEE may rescue cognitive decline in SAMP8 mice, possibly via the amelioration of biochemical pathologies as supported by our *in vitro* results.

The process of neurogenesis occurs mainly in two neurogenic niches: the dentate gyrus (DG) of the hippocampus and the subventricular zone (SVZ) of the lateral ventricles (Spalding et al., 2013; Aimone et al., 2014). These niches have neural stem/progenitor cell pools within which cells divide and generate neurons (and glia), and decades of studies have established that new neurons are continually produced in these two locations in the mammalian brain throughout adulthood. Although a recent study suggested persistent adult hippocampal neurogenesis in human (Boldrini et al., 2018), not all studies have found evidence for human hippocampal neurogenesis in adults (Sorrells et al., 2018), likely caused by differences in immunohistochemical parameters such as post-mortem handling of tissue (Flor-García et al., 2020). Neurogenic capacity declines with age, exacerbated in neurodegenerative diseases such as AD (Lazarov and Marr, 2010; Lee et al., 2012; Winner and Winkler, 2015; Trinchero et al., 2017; Moreno-Jiménez et al., 2019). Approaches to improve neurogenic defects have been studied as a therapeutic target, and the effect of dietary polyphenols on promoting neurogenesis has been suggested as a potential therapeutic candidate (Phillips, 2017). In this study, given the association between AHN and spatial memory, we performed BrdU labeling to examine AHN. The total number of BrdU^+^ cells was unchanged among all three groups: SAMR1 and SAMP8 controls, as well as STEE-fed SAMP8 mice. We, therefore, examined changes in newborn neurons (BrdU^+^DCX^+)^ or astrocytic precursor cells (BrdU^+^GFAP^+^) in the SGZ of SAMP8 mice. Our findings suggest that even the subtle increase of the newborn neuron population by about 60% may potentially be biologically relevant as STEE treatment could restore behavioral loss of SAMP8 mice in the MWM task. Plausibly, this increase in new immature neurons might be more pronounced if, for instance, STEE was given over a longer period or at higher doses. Further studies should address optimal dosing for memory and neurogenic enhancement.

The potential of STEE to induce neurogenesis is more strongly supported by our *in vitro* studies with hNSC. Indeed, the myriad effects of STEE *in vitro*, as well as *in vivo*, suggest a number of candidate cellular mechanisms by which it could rescue age-related cognitive decline. Neural stem cell fate and development are regulated by several factors, among which bHLH transcription factors play an important role in proliferation and differentiation. Ascl1 (also known as Mash1) is one of the bHLH factors, and is expressed in transit-amplifying cells, which proliferate and soon generate neuroblasts (Kim et al., 2011; Andersen et al., 2014; Sueda et al., 2019). On the other hand, repressor-type bHLH factors, such as Hes1, lead to suppression of Ascl1 and contribute to maintaining the quiescence of the cells. In this context, downregulated *HES1* expression in the cultures with STEE at the early differentiation stage corresponds to increased *ASCL1* expression and subsequent decrease of stem cell quiescence in hNSC. This can also be correlated with the downregulation of nestin expression in neurospheres cultured with STEE. These findings provoke interest in potential modulation of signaling pathways such as Notch by bHLH (Mizutani et al., 2007; Imayoshi et al., 2010; Andersen et al., 2014). Downregulation of *Jag1*, one of the cell surface ligands of Notch receptors, in STEE-fed mouse brain also raises interest. Moreover, the increased percentage of Tuj1^+^ neuronal cells in the hNSC adherent culture corresponds well with the results of qRT-PCR analysis in neurospheres and immunocytochemical analysis at early culture stages. The significant upregulation of HuD-encoding *Elavl4* and Dicer-encoding *Dicer1* and the significant downregulation of Musashi-2-encoding *Msi2* in the cerebral cortex of STEE-fed mice indicate evoked differentiation of cortical NSCs by STEE (Sakakibara et al., 2001; Akamatsu et al., 2005; Kawase-Koga et al., 2010; Saurat et al., 2013). Moreover, the modulated genes such as *Tenm2*, *Sox4*, and *Shh* in STEE-fed SAMP8 mouse brain suggest the possible effect of the extract as a neurodevelopmental modulator *in vivo* (Kenzelmann et al., 2007; Dave et al., 2011; Kamachi and Kondoh, 2013). Similar expressions of *Tenm2*, *Sox4*, and *Shh* were also observed in SAMR1 mouse brain.

While our findings from the SAMP8 mouse model are seemingly in conflict with our *in vitro* results, it should be noted that our investigations are specific to the SAMP8 mouse and therefore do not rule out potential effects on other mouse models of dementia or with different doses of STEE. Critically, neurogenic deficits in the SAMP8 mouse model have been largely characterized in aged mice. Gang et al., for example, only saw a significant decrease in BrdU^+^ and DCX^+^ cells in 10-month old SAMP8 mice compared to age-matched SAMR1 controls (Gang et al., 2011). A separate study by Díaz-Moreno et al. reported reduced neurogenesis in 14-month old SAMP8 mice (Diaz-Moreno et al., 2013). Our study showed that STEE could reverse the memory deficits in SAMP8 mice in the MWM task at 4 months of age. Because the MWM task is hippocampal-dependent, we tested for the potential involvement of AHN in the rescue of spatial memory deficits in the STEE-fed mice. Our findings corroborated prior studies that showed neurogenic changes do not occur between SAMR1 mice and SAMP8 controls at this age. Although we cannot discard the relevance between the slightly increased AHN and the improved spatial learning and memory, further studies are warranted to examine potential positive effects of STEE in aged SAMP8 mice with more overt cognitive and neurogenic deficits. We found several effects of STEE *in vitro*; thus, it is plausible that STEE might influence a range of subtle cellular changes that cumulatively reverse the memory decline in SAMP8 mice. Notably, we found that STEE promoting spatial memory recovery is correlated with the restoration of cortical DA, NE, and ACh levels.

Regulation of monoamine levels in the brain is critical for maintaining cognitive function, and an imbalance of monoamine transmitters such as DA, NE, and 5-HT plays a major role in the onset and progression of cognitive disorders (Naoi et al., 2018). As well as catecholaminergic neurons, cholinergic neurons are widely distributed in the CNS. The cholinergic system is involved in critical physiological processes such as attention, learning and memory, and wakefulness (Ferreira-Vieira et al., 2016). Therefore, modulation of neurotransmitter levels has been suggested as a therapeutic target, as evidenced by the use of serotonin reuptake inhibitors (SSRIs) and acetylcholinesterase inhibitors (AChEIs) for the treatment of cognitive impairment (Recanatini and Valenti, 2004). Degeneration of catecholaminergic neurons (including dopaminergic and noradrenergic) seriously worsens in SAMP8 mice from 8 months on (Karasawa et al., 1997), and significant decrease of ACh level has been observed in 3-month old SAMP8 mice hippocampi and cortices (Zhang et al., 2017). Enhanced synaptic plasticity in the cerebral cortex of STEE-fed mice could be explained by the significant upregulation of *Mbp*, a major myelin protein and essential for saltatory nerve conduction, and of *Syt1*, an important regulator of synaptic vesicle transport (Fields, 2015; Schupp et al., 2016). *Mbp* and *Syt1* were also upregulated in SAMR1 mice compared to SAMP8 controls. Additionally, the upregulation of phagocytic receptor *Mertk* and somatic Ca^2+^ mediator *Itpr2* may suggest developmental synapse pruning and remodeling by astrocytes in the brain of STEE-fed mice (Chung et al., 2013; Yang et al., 2016). Restored cortical neurotransmission in SAMP8 mice by STEE administration might contribute to the behavioral improvement of mice in the MWM task; however, the MWM task has an aspect that depends on hippocampal brain function. In this context, there is room for investigation on changes in hippocampal neurotransmitter levels, which is also important to neurogenesis (Lazarov and Marr, 2010; Winner and Winkler, 2015; Naoi et al., 2018).

In addition to our *in vivo* work, SH-SY5Y cells were used for *in vitro* assays. This cell line is known to have catecholaminergic neuron characteristics including expression of cholinergic (and dopaminergic) neuronal markers (Kume et al., 2008; Kovalevich and Langford, 2013). Our experimental results obtained from SH-SY5Y cultures showed that STEE stimulated neuronal cell metabolic activity through upregulation of glycolysis evidenced by increased transcript levels of *PGK1*, *PGAM1*, *PKM* and *PC* in the cells. Mitochondrial dysfunction is characterized as one of the biological processes accompanying aging and its associated cognitive decline, and is linked to decreased antioxidant defenses due to high energy demands (Grimm and Eckert, 2017). As an approach to enhance mitochondrial activity and reduce cognitive decline, the antioxidant properties of phytochemicals have received significant attention. For example, anthocyanin, which is a naturally occurring potent antioxidant, has been reported to improve spatial memory and restore brain ATP levels *in vivo* (Andres-Lacueva et al., 2005; Gutierres et al., 2012). Recent studies have shown that increasing glycolysis by PGK1 activation slows neurodegeneration in Parkinson’s disease (Cai et al., 2019). PGAM1, in turn, is reported to be protective against neuronal damage from oxidative stress or ischemia (Kim et al., 2020). Another protein we studied, PKM is one of the rate-limiting enzymes in the glycolytic reaction, and finally, PC connects glycolysis and the tricarboxylic acid (TCA) cycle through its catalytic action. The rapid increase of oxidative stress in SAMP8 mice is evidenced as elevated levels of oxidative stress markers such as lipid peroxide in the cerebral cortex of 4−8 weeks old young SAMP8 mice compared to SAMR1 controls (Sato et al., 1996). The present study suggests that STEE can promote glucose metabolism in neuronal cells, which in turn can counter age-related neuronal oxidative damage and reduced neurotransmission. Also, neurons prioritize lactate-uptake over glucose to sustain their oxidative demands; therefore, supply of lactate mediated through astrocytic glycolysis is thought to be critically important for maintaining neuronal activity and memory formation (Suzuki et al., 2011; Alberini et al., 2018). Indeed, the upregulation of pyruvate dehydrogenase kinases (PDKs), *Pdk1* and *Pdk3*, and a monocarboxylate transporter-encoding *Slc16a4 in vivo* may indicate active production and transport of lactate in STEE-fed mice. (Tadi et al., 2015). *Pdk1* and *Slc16a4* were also upregulated in SAMR1 mice compared to SAMP8 controls. Although the present study with hNSCs suggests that STEE might contribute to astrocytic process lengthening, the association between cellular energy metabolism and morphogenesis is uncertain. However, the findings would encourage the investigation of the effects of STEE on astrocyte function, including their glucose metabolism.

Microarray analysis showed the increased expression of *Ntrk2*, specifically in STEE-fed mice brain when compared to SAMP8 control. *Ntrk2* encodes tropomyosin receptor kinase B (TrkB), which is a receptor for neurotrophins such as brain-derived neurotrophic factor (BDNF). TrkB has several isoforms, among them, TrkB-FL is a full-length receptor, and TrkB-T1 is a truncated subtype. TrkB-FL signaling contributes to BDNF signal transduction as well as to nervous system development, including myelination and cell survival, through several classical pathways such as the PI3K/Akt pathway (Cosgaya et al., 2002; Gupta et al., 2013; Tejeda and Diaz-Guerra, 2017). TrkB-T1 transduces the Rho signaling pathway and regulates cellular morphogenesis or Ca^2+^ influx (Rose et al., 2003; Ohira et al., 2007). We also found that STEE treatment upregulated *Pik3r1* and *Limk1* expressions. *Pik3r1* is the predominant regulatory isoform of PI3K and encodes p85α subunit of PI3K. LIMK1 is activated downstream of Rho signaling and plays an essential role in synaptic transmission, plasticity, and memory formation. Therefore, it may be anticipated that the observed neurodevelopmental effects of STEE in this study were mediated by the activation of TrkB and Rho signaling evident by the upregulation of their downstream effectors *Pik3r1* and *Limk1*, respectively (Gupta et al., 2013; Tejeda and Diaz-Guerra, 2017; Ravindran et al., 2019). Particularly, recently explored role of astrocytic TrkB-T1 signaling in astrocyte morphogenesis (Holt et al., 2019) warrants further research on the effect of STEE and its active compounds on TrkB activation.

Chemical analysis revealed CQA derivatives as the major polyphenols in the sugarcane top. CQA and its derivatives are broadly distributed phytochemicals in plants and their health benefits are widely investigated. Previous studies have reported that CQA and its derivatives have antibacterial, anticancer, antihyperglycemic, and neuroprotective properties (Kimura et al., 1985; Yoshimoto et al., 2002; Matsui et al., 2004; Miyamae et al., 2011a). Our previous studies have reported that CQA derivatives could activate mitochondrial ATP production mediated through energy metabolism promotion characterized by the upregulation of glycolytic enzymes (Han et al., 2010; Miyamae et al., 2011a). Also, Ferulic acid, one of the cinnamic acid derivatives, has shown antidepressant-like effects modulated by the increased expression of glycolytic genes, including *PKM* and *PC* and by increased monoamine levels in the mouse brain limbic system (Sasaki et al., 2019b). Moreover, our recent study has suggested that CQA derivative increases G0/G1 cell cycle arrest in hNSC and leads to its differentiation (Sasaki et al., 2019a). Considering the facts mentioned above, we can postulate that the CQAs in the sugarcane top extract may act as the glycolysis up-regulators and neural stem cell fate regulators observed in the present study. An ultra-high-performance liquid chromatography tandem mass spectrometry study by Su et al. has demonstrated that several mono-CQA and di-CQA isomers can pass through the blood-brain barrier (BBB) (Su et al., 2014). Although in our study, we have not explicitly investigated the effect of STEE on endothelial cells of the BBB, we found that STEE induced astrocytic process lengthening in hNSCs, and decreased expression of *Tnfrsf25*, *Vcam1*, and *Mmp9* in mice cortex, suggesting that STEE may improve age-associated degeneration of BBB properties (Wosik et al., 2007). ISO, a natural flavonoid, is a luteolin glycoside as shown in its chemical identification name- luteolin-6-C-glucoside. ISO exists in several plants such as rooibos (*Aspalathus Linearis*) (Breiter et al., 2011), and has been reported to exhibit a variety of bioactivities, including antioxidant, anti-inflammatory, and anticancer effects (Tunalier et al., 2007; Yuan et al., 2013, 2016). Furthermore, a previous study has reported that ISO acts as an ATP non-competitive glycogen synthase kinase-3β (GSK3β) inhibitor and alleviates tau phosphorylation and Aβ toxicity (Liang et al., 2016). Therefore, this luteolin glycoside may also contribute to oxidative stress reduction and mitochondrial activation by enhancing cellular oxidative metabolism (Theeuwes et al., 2017; Yang et al., 2017). Also, GSK3β inhibition can induce neural stem cell proliferation and neurogenesis (Morales-Garcia et al., 2012), suggesting potential effects of ISO on neurogenesis. Daily intake of coffee or rooibos has not been reported to cause any serious damage to health. Additionally, several plant extracts, such as *Ilex guayusa*, rich in mono- and di-CQA derivatives have been approved by the FDA as GRAS (generally regarded as safe; GRAS Notice No. GRN 000869). Also, Amano et al. have reported that minor gastrointestinal side effects can be observed only in very high dose of 5-CQA (>100 μM), whereas 1 g of STEE has approximately 13.86 μM of 5-CQA (Amano et al., 2019). Therefore, daily intake of these polyphenols is unlikely to cause any serious side effects. In addition to the major peaks, several other peaks were also observed in the chromatogram. Analysis of those minor peaks may lead to identification of further interesting phytochemicals in the extract. However, previous studies on the bioactivities of the compounds identified as the major constituents of STEE in the present study suggest that the extract from this freely available and plentiful biological resource is a promising nutrient source having a unique phytochemical make-up and showing physiological activity on several aspects of brain function.

Our previous study has shown that caffeoylquinic acid-rich plant extract ameliorated cognitive decline in SAMP8 mice but did not affect behavioral activities in SAMR1 mice (Sasaki et al., 2013). Therefore, in the present study, we did not include the STEE-fed SAMR1 group. This is supported by the literature, whereby SAMP8 investigations typically include the SAMP8 and SAMR1 controls without SAMR1 + treatment group (Yang et al., 2020).

## Conclusion

In conclusion, our data is the first to demonstrate that STEE ameliorates age-related cognitive function likely through modulation of neuronal energy metabolism and neural differentiation from neural stem/progenitor cells. In the present study, we used a single concentration of STEE for the *in vivo* experiments; therefore, further careful studies with multiple sample concentrations are required to reveal optimal dosage for the induction of neural activity *in vivo*. One of the advantages of STEE is that they contain multiple bioactive compounds that can target multiple pathologies simultaneously, and therefore, can be more efficacious than traditional drugs for age-related impairment of brain functions. The findings of this study suggest the potential of STEE as a novel nutritional intervention or nutraceutical for cognitive health.

## Data Availability Statement

All datasets generated for this study are included in this article/Supplementary Material. Microarray data are deposited in the NCBI Gene Expression Omnibus (GEO) under accession number: GSE151727 (https://www.ncbi.nlm.nih.gov/geo/query/acc.cgi?acc=GSE151727).

## Ethics Statement

All animal procedures were performed according to the guidelines of the Council of Physiological Society, Japan. Experimental protocols were approved by the Ethics Animal Care and Use Committee of University of Tsukuba, Japan.

## Author Contributions

KI and QW investigated the data, performed the methodology, carried out the formal analysis and data curation, wrote the original draft of the manuscript, and visualized the data. FF performed the software, carried out the formal analysis, and wrote, reviewed, and edited the manuscript. KS and KT investigated the data, performed the methodology, and validated the data. YA and HU contributed to conceptualization and resources. FS and HI contributed to conceptualization, resources, wrote, reviewed, and edited the manuscript, supervised the data, and carried out the project administration and funding acquisition. All the authors made substantial contributions to this article and approved the final article.

## Conflict of Interest

KI, HU, and YA are employed by the Nippo Co., Ltd. The remaining authors declare that the research was conducted in the absence of any commercial or financial relationships that could be construed as a potential conflict of interest.

## Footnotes

1 http://www.affymetrix.com

2 https://www.gsea-msigdb.org/gsea/index.jsp

3 http://software.broadinstitute.org/Morpheus

4 http://bioinformatics.psb.ugent.be/webtools/Venn/

5 https://www.ncbi.nlm.nih.gov/geo/info/linking.html
