# Supplementary material for: Sugarcane (Saccharum officinarum L.) Top Extract Ameliorates Cognitive Decline in Senescence Model SAMP8 Mice: Modulation of Neural Development and Energy Metabolism
Source: Front Cell Dev Biol. 2020 Oct 6;8:573487. doi: 10.3389/fcell.2020.573487 (PMC7573230; doi:10.3389/fcell.2020.573487)
Supplement: Supplementary file 1 [file Data_Sheet_1.ZIP › Supplementary_Material.docx]

Supplementary Material

## Supplementary Figures and Tables

**
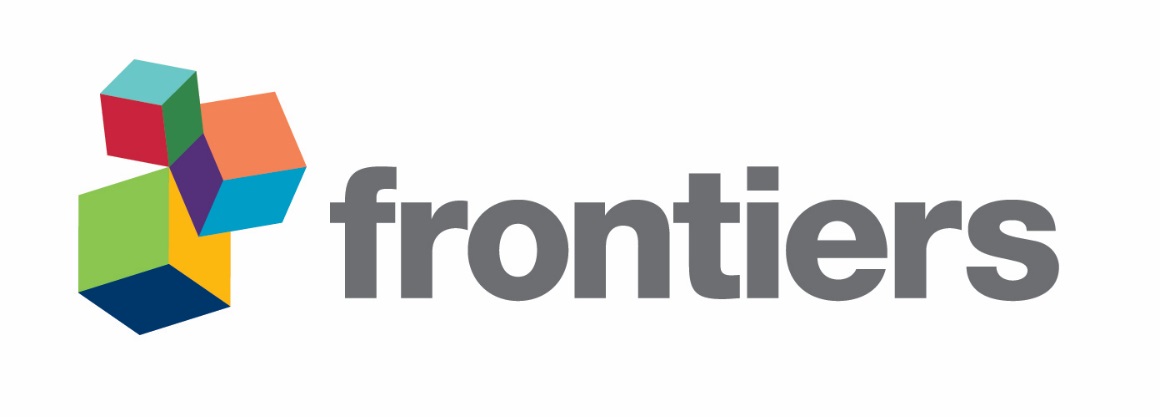
**

**Supplementary Figure 1** | Distribution of fold changes in mRNA expression levels between **(A)** SAMP8 control and SAMR1 mice, and **(B)** STEE-fed SAMP8 and SAMP8 control.

**Supplementary Figure 2** | Schematic diagram for the possible mechanism of the effect of STEE in the brain. STEE favors glucose metabolism, synaptic transmission, and neurodevelopment by inducing TrkB-mediated signal transduction. TrkB-FL or TrkB-T1 activated by STEE or its compounds trigger signaling such as PI3K/Akt pathway or Rho signaling pathway, resulting in transcription of genes related to above biological outcome.

**Supplementary Table 1 |** Characteristics of genes presented in the heatmap of Figure 6B

**Supplementary Table 2 |** Characteristics of genes presented in the heatmap of Figure 6C

**Supplementary Figure 3 |** Effects of STEE on SH-SY5Y cells. **(A)** Effect of different concentrations of STEE on the MTT assay in SH-SY5Y cells. The cells were treated with 1, 5, 10, 25, and 50 µg/mL of STEE for 72 hrs. The MTT enzymatic product was measured as optical density (OD) at 570 nm. Results are expressed as relative to control percentages and presented as mean ± *SD*. Comparisons were performed by one-way ANOVA with Dunnett’s post-hoc test: ***p* < 0.01. **(B)** Effect of STEE (50 µg/mL) on the total viable cell number. The cell numbers were determined at different time points (0, 12, 24, 48, and 72 hrs) after STEE treatment by ViaCount assay with flow cytometry. Values are presented as mean ± *SD*. Comparisons were performed using two-way ANOVA with Sidak’s post-hoc test.
