# Supplementary material for: Sugarcane (Saccharum officinarum L.) Top Extract Ameliorates Cognitive Decline in Senescence Model SAMP8 Mice: Modulation of Neural Development and Energy Metabolism
Source: Front Cell Dev Biol. 2020 Oct 6;8:573487. doi: 10.3389/fcell.2020.573487 (PMC7573230; doi:10.3389/fcell.2020.573487)
Supplement: Supplementary file 1 [file Data_Sheet_1.ZIP › Supplementary Table 1_Revised.pdf]

Gene Symbol	Description	Fold Change (P8 control vs R1)	Fold Change (P8 + STEE vs P8 control)	Biological Process# (GO ID)
Tenm4	Teneurin transmembrane protein 4	− 1.06	1.12*	Signal transduction (GO:0007165); Multicellular organism development (GO:0007275); Cell differentiation (GO:0030154)
Traf6	TNF receptor-associated factor 6	− 1.06†	1.12*	Activation of MAPK activity (GO:0000187); Protein polyubiquitination (GO:0000209)
Ntn5	Netrin 5	−1.11	1.18*	Nervous system development (GO:0007399); Motor neuron axon guidance (GO:0008045)
Prkd1	Protein kinase D1	− 1.11	1.16*	Immune system process (GO:0002376); Protein phosphorylation (GO:0006468); Apoptotic process (GO:0006915)
Gpm6b	Glycoprotein m6b	− 1.19	1.26*	Nervous system development (GO:0007399); Cell differentiation (GO:0030154)
Slc16a4	Solute carrier family 16 (monocarboxylic acid transporters), member 4	−1.17†	1.24*	Monocarboxylic acid transport (GO:0015718); Transmembrane transport (GO:0055085)
Tnik	TRAF2 and NCK interacting kinase	− 1.39*	1.48*	Protein phosphorylation (GO:0006468); Cytoskeleton organization (GO:0007010); Nervous system development (GO:0007399)
Smad6	SMAD family member 6	− 1.1†	1.11*	Outflow tract septum morphogenesis (GO:0003148)
Sdc4	Syndecan 4	− 1.19†	1.2*	Neural tube closure (GO:0001843)
Mbp	Myelin basic protein	− 1.24†	1.23*	Chemical synaptic transmission (GO:0007268); Central nervous system development (GO:0007417)
Itpr2	Inositol 1,4,5-trisphosphate receptor type 2	− 1.13	1.11*	Calcium ion transport (GO:0006816); Signal transduction (GO:0007165)
Syt1	Synaptotagmin I	− 1.31*	1.27*	Chemical synaptic transmission (GO:0007268); Neurotransmitter secretion (GO:0007269); Brain development (GO:0007420); Glutamate secretion (GO:0014047)
Pdk1	Pyruvate dehydrogenase kinase, isoenzyme 1	− 1.36*	1.22*	Glucose metabolic process (GO:0006006); Protein phosphorylation (GO:0006468); Cell proliferation (GO:0008283)
Tenm2	Teneurin transmembrane protein 2	− 1.55†	1.34*	Cell adhesion (GO:0007155); Signal transduction (GO:0007165); Neuron development (GO:0048666)
Limk1	LIM-domain containing, protein kinase	− 1.29*	1.16*	Protein phosphorylation (GO:0006468); Signal transduction (GO:0007165); Rho protein signal transduction (GO:0007266); Nervous system development (GO:0007399)
Bhlhe22	Basic helix-loop-helix family, member e22	− 1.25	1.12*	Nervous system development (GO:0007399); Neurogenesis (GO:0022008)
Tnfrsf25	Tumor necrosis factor receptor superfamily, member 25	1.16	− 1.56*	Apoptotic process (GO:0006915); Tumor necrosis factor-mediated signaling pathway (GO:0033209); Regulation of apoptotic process (GO:0042981)
Map2k3	Mitogen-activated protein kinase kinase 3	1.12	− 1.29*	Activation of MAPK activity (GO:0000187); Protein phosphorylation (GO:0006468); Inflammatory response (GO:0006954); Signal transduction (GO:0007165)
Crkl	V-crk sarcoma virus CT10 oncogene homolog (avian)-like	1.06	− 1.13*	Activation of MAPK activity (GO:0000187); Regulation of cell growth (GO:0001558)
Gadd45b	Growth arrest and DNA-damage-inducible 45 beta	1.29*	− 1.7*	Negative regulation of protein kinase activity (GO:0006469); Apoptotic process (GO:0006915); Multicellular organism development (GO:0007275)
Lgr5	Leucine rich repeat containing G protein coupled receptor 5	1.14†	− 1.26*	G protein-coupled receptor signaling pathway (GO:0007186); Adenylate cyclase-activating G protein-coupled receptor signaling pathway (GO:0007189)
Gfap	Glial fibrillary acidic protein	1.12	− 1.17*	Positive regulation of Schwann cell proliferation (GO:0010625); Negative regulation of neuron projection development (GO:0010977); Astrocyte development (GO:0014002)
Sox4	SRY-box containing gene 4	1.08†	− 1.1*	Skeletal system development (GO:0001501); Neural tube formation (GO:0001841)
Pdgfb	Platelet derived growth factor, B polypeptide	1.3	− 1.43*	MAPK cascade (GO:0000165); Platelet degranulation (GO:0002576)
Smad1	SMAD family member 1	1.15†	− 1.18*	Signal transduction (GO:0007165); Transforming growth factor beta receptor signaling pathway (GO:0007179); SMAD protein complex assembly (GO:0007183)
Shh	Sonic hedgehog	1.12*	− 1.12*	Negative regulation of transcription by RNA polymerase II (GO:0000122); Angiogenesis (GO:0001525)
Acvr1	Activin A receptor, type 1	1.22*	− 1.17*	G1/S transition of mitotic cell cycle (GO:0000082); Angiogenesis (GO:0001525)

*:Fold change satisfied with $p < 0.05$; †:Fold change satisfied with $0.05 < p < 0.1$; #:Biological process is obtained from GeneCards® database (https://www.genecards.org/).
