# Supplementary material for: Sugarcane (Saccharum officinarum L.) Top Extract Ameliorates Cognitive Decline in Senescence Model SAMP8 Mice: Modulation of Neural Development and Energy Metabolism
Source: Front Cell Dev Biol. 2020 Oct 6;8:573487. doi: 10.3389/fcell.2020.573487 (PMC7573230; doi:10.3389/fcell.2020.573487)
Supplement: Supplementary file 1 [file Data_Sheet_1.ZIP › Supplementary Table 2_Revised.pdf]

| Gene Symbol | Description | Fold Change (P8 control vs R1) | Fold Change (P8 + STEE vs P8 control) | Biological Process# (GO ID) |
|---|---|---|---|---|
| *Rnd1* | Rho family GTPase 1 | − 1.1 | 1.31* | Actin filament organization (GO:0007015); Small GTPase mediated signal transduction (GO:0007264); Rho protein signal transduction (GO:0007266) |
| *Elavl4* | ELAV (embryonic lethal, abnormal vision, Drosophila)-like 4 (Hu antigen D) | − 1.02 | 1.21* | Learning (GO:0007612); Locomotory behavior (GO:0007626); Dendrite morphogenesis (GO:0048813) |
| *Dicer1* | Dicer 1, ribonuclease type III | 1.01 | 1.12* | RNA processing (GO:0006396); miRNA metabolic process (GO:0010586) |
| *Cd34* | CD34 antigen | 1.06 | 1.21* | Endothelial cell proliferation (GO:0001935); Endothelium development (GO:0003158); Cell adhesion (GO:0007155) |
| *Mertk* | C-mer proto-oncogene tyrosine kinase | 1.1* | 1.3* | Protein phosphorylation (GO:0006468); Phagocytosis (GO:0006909); Cell surface receptor signaling pathway (GO:0007166) |
| *Sema3a* | Sema domain, immunoglobulin domain (Ig), short basic domain, secreted, (semaphorin) 3A | 1.05† | 1.1* | Neural crest cell migration (GO:0001755); Neuron migration (GO:0001764) |
| *Ntrk2* | Neurotrophic tyrosine kinase, receptor, type 2 | 1.14* | 1.18* | Neuron migration (GO:0001764); Positive regulation of protein phosphorylation (GO:0001934); Transmembrane receptor protein tyrosine kinase signaling pathway (GO:0007169) |
| *Pdk3* | Pyruvate dehydrogenase kinase, isoenzyme 3 | 1.15† | 1.17* | Glucose metabolic process (GO:0006006); Regulation of acetyl-CoA biosynthetic process from pyruvate (GO:0010510); Regulation of glucose metabolic process (GO:0010906) |
| *Mapk11* | Mitogen-activated protein kinase 11 | 1.19† | 1.14* | Activation of MAPK activity (GO:0000187); Protein phosphorylation (GO:0006468); Ras protein signal transduction (GO:0007265); Regulation of gene expression (GO:0010468) |
| *Pik3r1* | Phosphatidylinositol 3-kinase, regulatory subunit, polypeptide 1 (p85 alpha) | 1.16* | 1.11* | Cellular glucose homeostasis (GO:0001678); Protein phosphorylation (GO:0006468); Protein import into nucleus (GO:0006606); Phosphatidylinositol biosynthetic process (GO:0006661) |
| *Vcam1* | Vascular cell adhesion molecule 1 | 1.03 | − 1.13* | Acute inflammatory response (GO:0002526); Chronic inflammatory response (GO:0002544); Cell adhesion (GO:0007155) |
| *Mmp9* | Matrix metallopeptidase 9 | 1.01 | − 1.11* | Proteolysis (GO:0006508); Cytokine-mediated signaling pathway (GO:0019221) |
| *Nodal* | Nodal | − 1.01 | − 1.11* | Negative regulation of transcription by RNA polymerase II (GO:0000122) |
| *Ccr5* | Chemokine (C-C motif) receptor 5 | − 1.05 | − 1.26* | MAPK cascade (GO:0000165); Dendritic cell chemotaxis (GO:0002407); Defense response (GO:0006952) |
| *Msi2* | Musashi RNA-binding protein 2 | − 1.03 | − 1.22* | Stem cell development (GO:0048864) |
| *Ptch1* | Patched homolog 1 | − 1.05 | − 1.2* | Negative regulation of transcription by RNA polymerase II (GO:0000122); Cell fate determination (GO:0001709); Neural tube formation (GO:0001841) |
| *Bcl2* | B cell leukemia/lymphoma 2 | − 1.08 | − 1.16* | Protein polyubiquitination (GO:0000209); Cell morphogenesis (GO:0000902) |
| *Jag1* | Jagged 1 | − 1.07 | − 1.1* | Cell fate determination (GO:0001709); Negative regulation of cell-matrix adhesion (GO:0001953) |

*:Fold change satisfied with $p < 0.05$; †:Fold change satisfied with $0.05 < p < 0.1$; #:Biological process is obtained from GeneCards® database (https://www.genecards.org/).
