# Supplementary figures and images for: Sugarcane (Saccharum officinarum L.) Top Extract Ameliorates Cognitive Decline in Senescence Model SAMP8 Mice: Modulation of Neural Development and Energy Metabolism
Source: Front Cell Dev Biol. 2020 Oct 6;8:573487. doi: 10.3389/fcell.2020.573487 (PMC7573230; doi:10.3389/fcell.2020.573487)

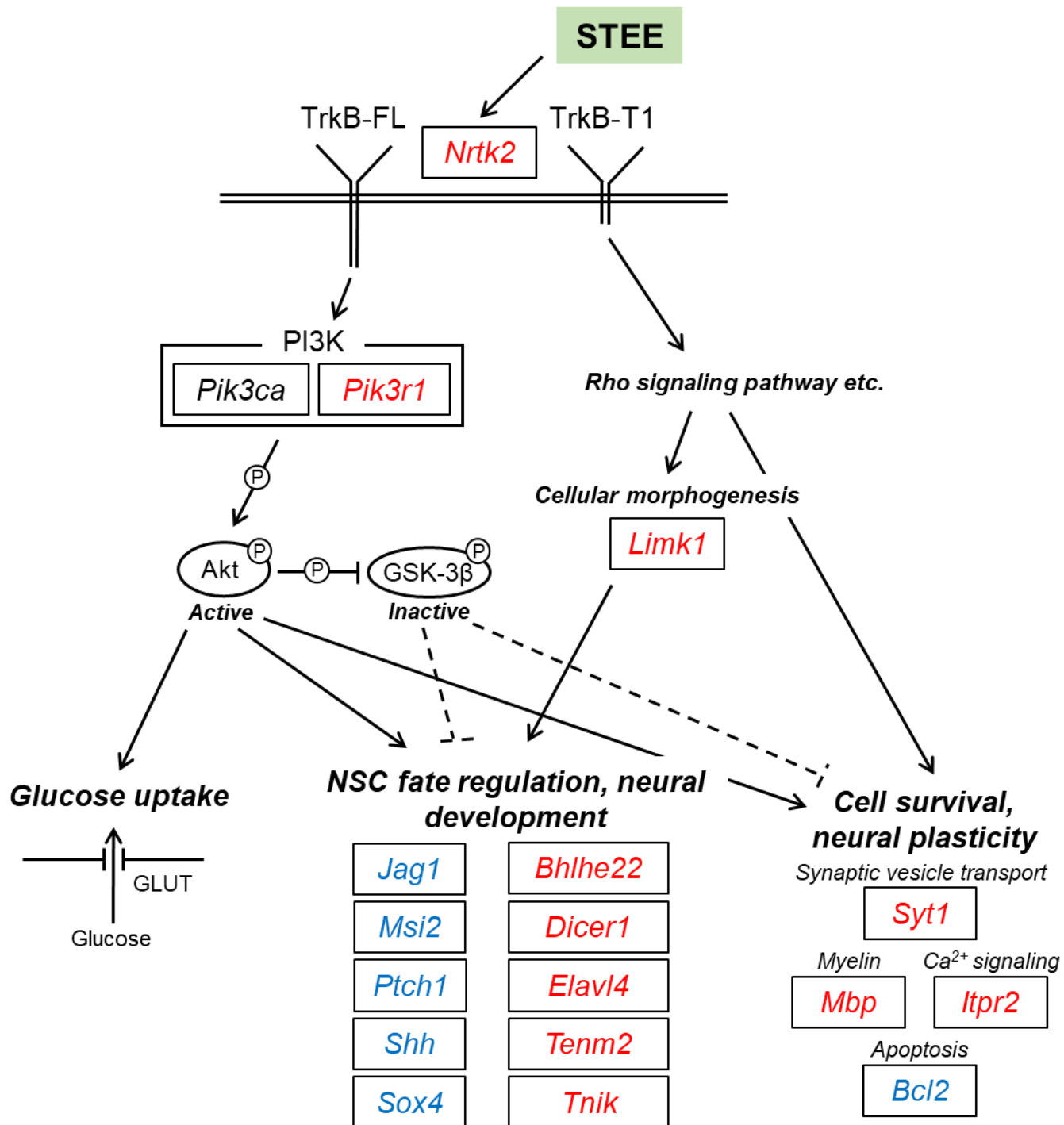

Supplement: Supplementary file 1 [file Data_Sheet_1.ZIP › Supplementary Figure 2.pdf]

(A)

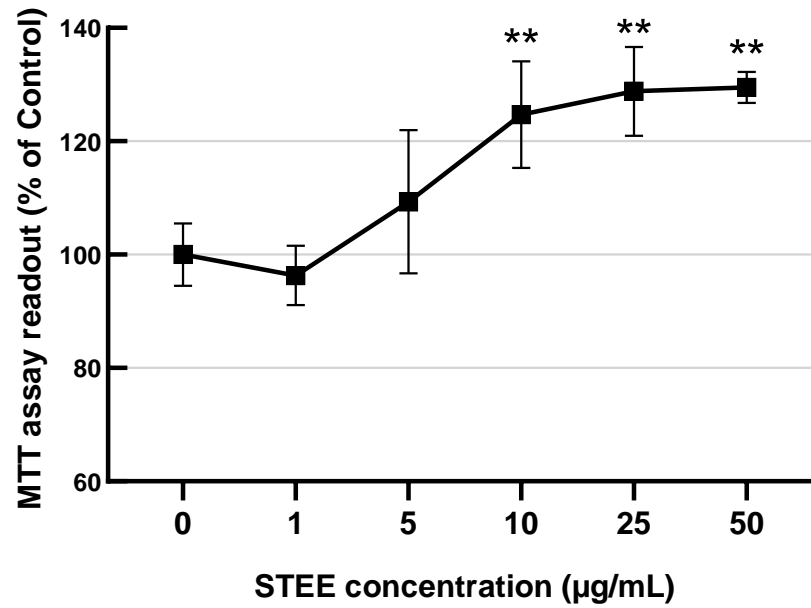

(B)

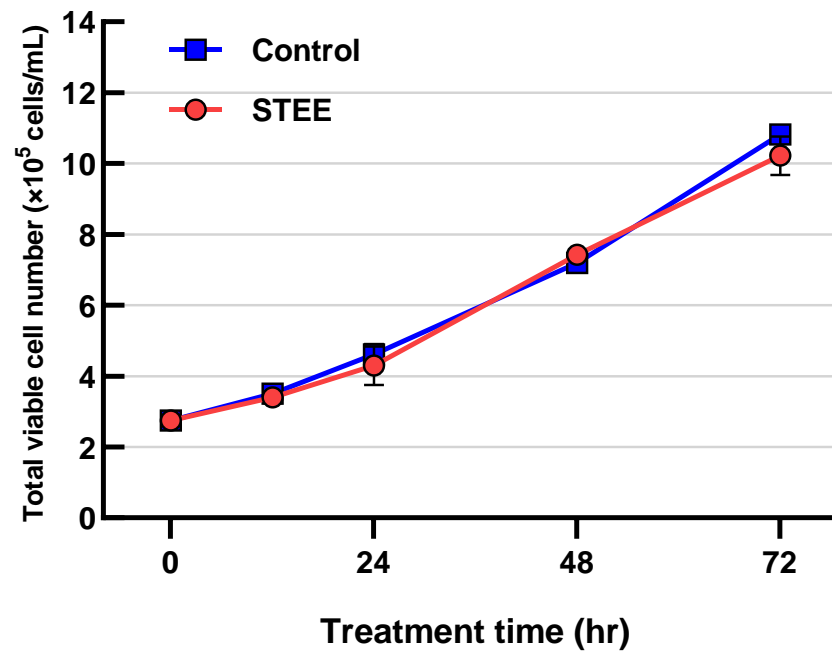

Supplement: Supplementary file 1 [file Data_Sheet_1.ZIP › Supplementary Figure 3.pdf]

(A)

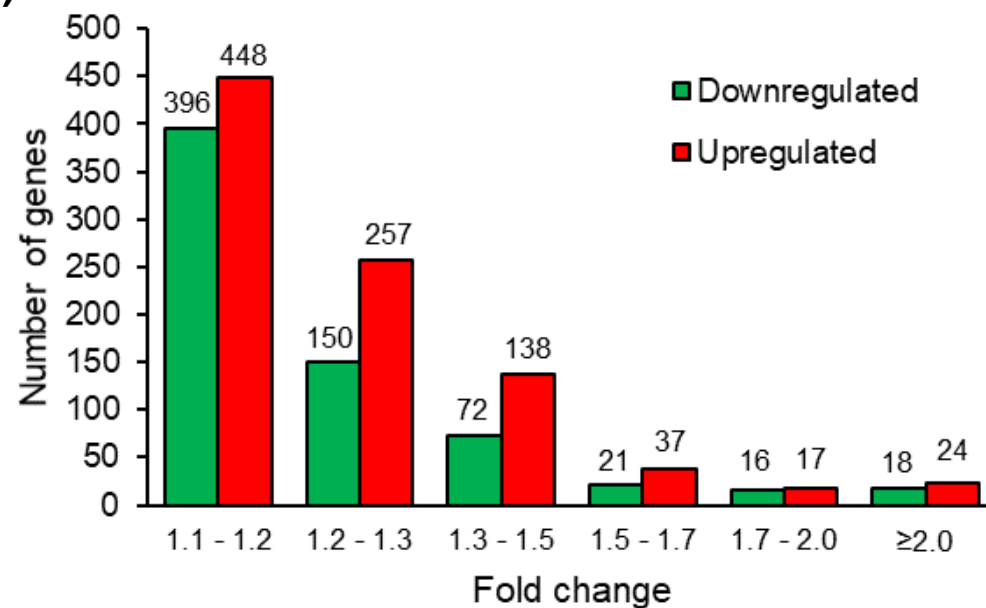

(B)

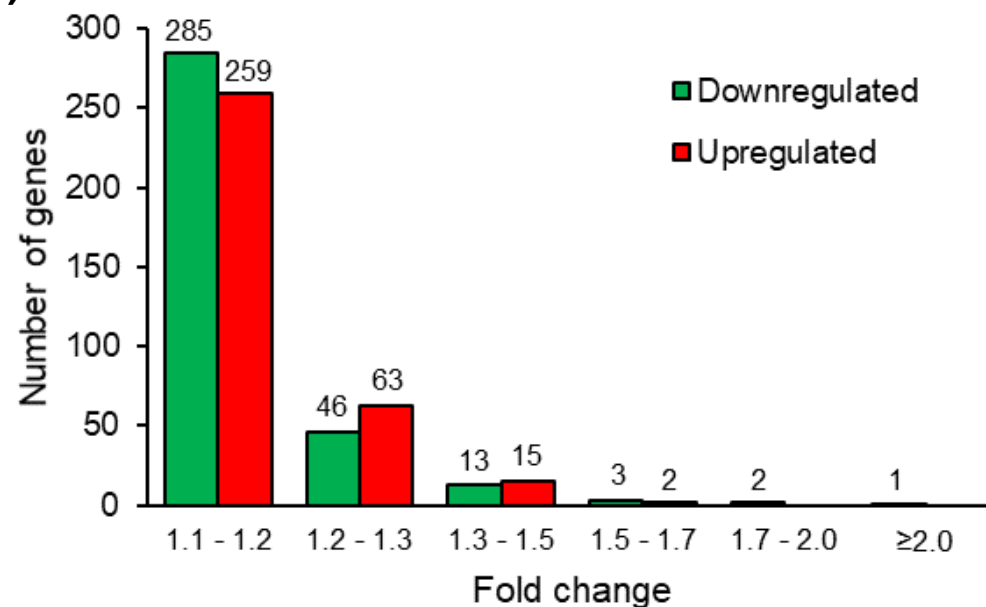

Supplement: Supplementary file 1 [file Data_Sheet_1.ZIP › Supplementary Figure 1.pdf]
